# The LRRK2 G2019S variant rewires Rab GTPase phosphorylation after lysosomal damage and promotes cell death in human macrophages

**DOI:** 10.1242/jcs.265083

**Published:** 2026-07-27

**Authors:** Rebecca Morrison, Simeon R. Mihaylov, Chak Hon Luk, Cyril Lemerle, Angela Rodgers, Natalia Athanasiadi, Huw R. Morris, Enrica Pellegrino, Sila K. Ultanir, Maximiliano G. Gutierrez

**Affiliations:** ^1^ Host-Pathogen Interactions in Tuberculosis Laboratory, The Francis Crick Institute, 1 Midland Road, London NW1 1AT, UK; ^2^ Kinases and Brain Development Laboratory, The Francis Crick Institute, 1 Midland Road, London NW1 1AT, UK; ^3^ Department of Clinical and Movement Neurosciences, UCL Queen Square Institute of Neurology, UCL Movement Disorders Centre, University College London, London WC1N 3BG, UK

**Keywords:** LRRK2, Rab GTPase, Lysosomal damage, Macrophage, Parkinson's disease, Apoptosis

## Abstract

Variants in leucine-rich repeat kinase 2 (LRRK2) are the most common genetic cause of Parkinson's disease, yet how these variants alter immune cell function remains unclear. Because LRRK2 is activated by lysosomal damage in macrophages, we investigated how the pathogenic G2019S variant affects macrophage responses to lysosomal damage. Here, we show that LRRK2 G2019S has an effect during lysosomal damage through kinase-dependent and kinase-independent mechanisms. Phosphoproteomic analysis revealed that lysosomal damage induces selective rewiring of LRRK2-dependent Rab GTPase phosphorylation, characterised by increased Rab12 phosphorylation and reduced Rab35 phosphorylation without global kinase hyperactivation. Strikingly, LRRK2 G2019S macrophages showed increased susceptibility to apoptosis following lysosomal damage. This increase in cell death occurred independently of the kinase activity, indicating a distinct kinase-independent role of LRRK2 in regulating cell survival. We generated isogenic induced pluripotent stem cells from patients carrying the LRRK2 G2019S variant and confirmed that LRRK2 G2019S macrophages are more susceptible to cell death in a kinase-independent manner. Together, our findings support a model in which the LRRK2 G2019S variant selectively changes the phosphorylation of Rab GTPases in macrophages and increases cell death after lysosomal damage in macrophages.

## Teaser

**Highlighted Article:** LRRK2 G2019S rewires Rab GTPase phosphorylation during lysosomal damage and promotes kinase-independent apoptosis in macrophages, linking Parkinson's disease genetics to immune cell dysfunction.

## INTRODUCTION

Variants in leucine-rich repeat kinase 2 (LRRK2) represent the most common genetic cause of Parkinson's disease (PD) (Ferreira and Massano, 2017; Paisan-Ruiz et al., 2004; Williams-Gray et al., 2006). Although LRRK2 has been studied extensively in neurons, increasing evidence indicates that it also functions in macrophages and microglia, where it regulates inflammatory responses and vesicular trafficking (Dzamko et al., 2012; Gardet et al., 2010; Hakimi et al., 2011; Kania et al., 2023; Lee et al., 2020; Oun et al., 2023; Tansey et al., 2022; Yan and Liu, 2017). In these cells, LRRK2 is closely associated with lysosomes, where it regulates lysosomal function (Yadavalli and Ferguson, 2023) and phosphorylates Rab GTPases in response to lysosomal damage (Abe et al., 2024; Bonet-Ponce et al., 2024; Herbst et al., 2020). However, how pathogenic LRRK2 variants alter immune cell responses to lysosomal damage remains poorly understood.

Macrophages are highly dynamic immune cells that experience recurrent lysosomal stress during phagocytosis and inflammatory responses (Katsnelson et al., 2016). Maintenance of lysosomal integrity is essential for cell survival, and damage to lysosomes triggers coordinated repair pathways that restore cellular homeostasis (Jia et al., 2020; Radulovic et al., 2018; Skowyra et al., 2018). Disruption of these processes can lead to cell death and contribute to inflammation and disease (Aits and Jaattela, 2013; de Castro et al., 2016; Wang et al., 2018). In the context of PD, lysosomal stress can be induced by pathogenic α-synuclein fibrils, resulting in activation of LRRK2, linking lysosomal damage in macrophages to PD-relevant trafficking responses (Abe et al., 2024).

The LRRK2 G2019S mutation, the most common PD-associated variant (Ferreira and Massano, 2017; Paisan-Ruiz et al., 2004; Williams-Gray et al., 2006), increases kinase activity and alters phosphorylation of Rab GTPases, key regulators of intracellular trafficking (Greggio et al., 2006; Karayel et al., 2020; Myasnikov et al., 2021; West et al., 2005). However, whether this variant leads to a global increase in kinase signalling or instead selectively rewires downstream pathways in immune cells remains unclear.

Here, we used primary mouse macrophages and human induced pluripotent stem cell (iPSC)-derived macrophages to investigate how LRRK2 G2019S alters cellular responses to lysosomal damage. We show that the LRRK2 G2019S variant selectively reshapes Rab GTPase phosphorylation rather than globally enhancing kinase activity. In parallel, we uncover a kinase-independent role for LRRK2 in regulating apoptosis that is disrupted by G2019S. These findings indicate that the pathogenic LRRK2 G2019S variant affects global Rab GTPase phosphorylation and impacts cell survival after lysosomal damage.

## RESULTS

### LRRK2 kinase activity selectively targets Rab GTPases after lysosomal damage

**Fig. 1.**
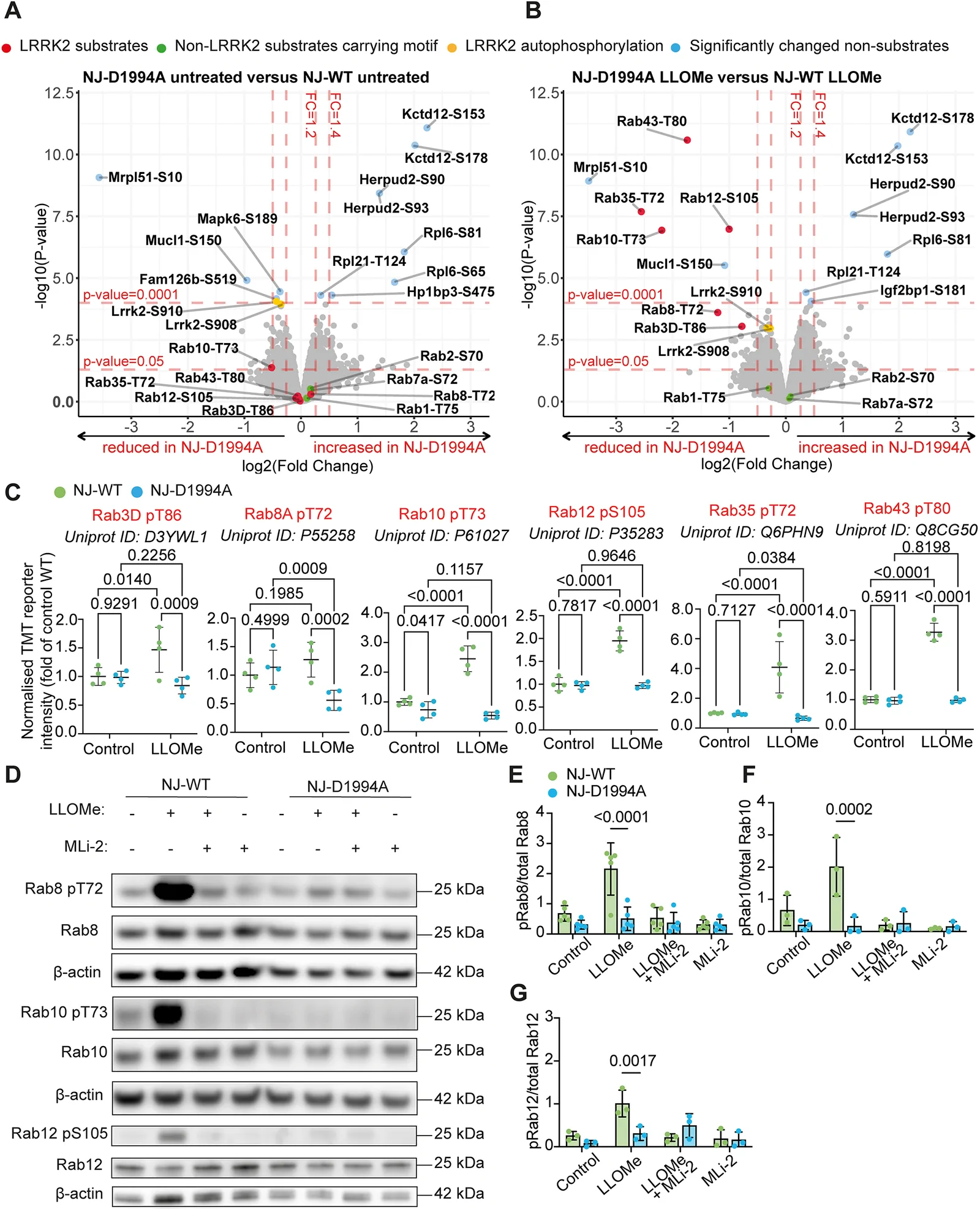
**LRRK2 kinase activity selectively targets Rab GTPases following lysosomal damage.** (A,B) NJ-WT and NJ-D1994A bone marrow-derived macrophages (BMDMs) were treated with 1 mM LLOMe for 30 min and cells were analysed by TMTpro 16-plex mass spectrometry. Volcano plots of the phosphoproteome in the untreated (A) and LLOMe-treated (B) conditions showing the difference in phosphorylation levels between NJ-WT and NJ-D1994A BMDMs. Each point represents one phosphorylation site of a protein. The *x*-axis shows the log_2_-transformed fold change (FC), and the *y*-axis shows the significance by −log_10_-transformed *P*-value, obtained by linear models for microarray data. FC>1.2 and *P*<0.05 were deemed significant. (C) Scatterplots showing the normalised tandem mass tag (TMT) reporter intensity data obtained by mass spectrometry and *P*-values for individual significantly altered Rab phosphosites. CONSTANd-normalised and log_2_-transformed raw TMT reporter intensities were back-transformed (2^x^) and expressed relative to the mean of the control wild-type (WT) group (set at 1). *n*=4 biological replicates per condition. *P*-values were calculated by linear models for microarray data (limma) statistics on the normalised log_2_-transformed data. Error bars represent s.d. (D) Western blot analysis of Rab8 pT72, Rab8, Rab10 pT73, Rab10, Rab12 pS105, Rab12 and β-actin in NJ-WT and NJ-D1994A macrophages in untreated, LLOMe, LLOMe+MLi-2 and MLi-2 conditions. (E–G) Rab8 pT72, Rab10 pT73 and Rab12 pS105 band intensities were quantified by densitometry and normalised to Rab8, Rab10 and Rab12 band intensities, respectively. Data are mean±s.d. from (E) five and (F,G) three independent experiments. Two-way ANOVA followed by Šidák's multiple comparisons test.

To define the substrates of LRRK2 that are activated during lysosomal damage in macrophages, we performed quantitative phosphoproteomics in murine bone marrow-derived macrophages (BMDMs) expressing either wild-type LRRK2 (NJ-WT) or a kinase-dead mutant (NJ-D1994A). Lysosomal damage was induced using L-leucyl-L-leucine methyl ester (LLOMe), a lysosomotropic agent that disrupts lysosomal membranes (Thiele and Lipsky, 1990). NJ-WT and NJ-D1994A macrophages showed similar levels of macrophage surface marker expression by flow cytometry, and no differences in lysosomal content, proteolytic activity or LRRK2 expression (Fig. S1A–I). Tandem mass tag (TMT)-labelled mass spectrometry of enriched phosphopeptides identified 29,076 phosphosites. When comparing the phosphosites in NJ-D1994A versus NJ-WT macrophages, significant changes [|log_2_(fold change or FC)|>1.2 and *P*<0.05] were observed in 396 sites (166 reduced and 230 increased) in the control group (Fig. 1A) and 577 sites (227 reduced and 350 increased) in the LLOMe-treated group (Fig. 1B). Comparative analysis revealed that lysosomal damage induced robust phosphorylation of a defined subset of Rab GTPases, including Rab3D, Rab8A (hereafter Rab8), Rab10, Rab12, Rab35 and Rab43, in a manner dependent on LRRK2 kinase activity (Fig. 1A–C). In contrast, Rab proteins lacking established LRRK2-dependent phosphorylation sites (Steger et al., 2017, 2016) showed no significant changes, indicating the specificity of this response (Fig. 1A,B; Fig. S2A–G). Notably, we did not detect substantial changes in non-Rab substrates following lysosomal damage, confirming that Rab GTPases represent the main targets of LRRK2 in this context (Fig. 1A,B; Fig. S2H–N).

Mass spectrometry analysis of the proteome identified 6530 proteins. When comparing the protein levels in NJ-D1994A versus NJ-WT macrophages, significant changes (|log_2_FC|>1.2 and *P*<0.05) were observed in 101 proteins (28 reduced and 73 increased) in the control group (Fig. S2A) and 94 proteins (37 reduced and 57 increased) in the LLOMe-treated group (Fig. S2B). Importantly, total protein levels of the Rab GTPases were unchanged, indicating that the observed differences reflect regulated phosphorylation rather than altered expression (Fig. S2A,B). Consistent with these findings, western blot analysis demonstrated that lysosomal damage increased the phosphorylation of Rab8, Rab10 and Rab12 in NJ-WT macrophages, and this response was abolished in NJ-D1994A cells harbouring kinase-dead LRRK2 and in cells treated with the LRRK2 inhibitor MLi-2 (Fig. 1D–G). Together, these results demonstrate that lysosomal damage activates LRRK2 kinase activity in macrophages and selectively drives phosphorylation of a defined subset of Rab GTPases, establishing Rab proteins as the primary downstream effectors of LRRK2 in macrophages.

### LRRK2 G2019S selectively rewires Rab phosphorylation following lysosomal damage

**Fig. 2.**
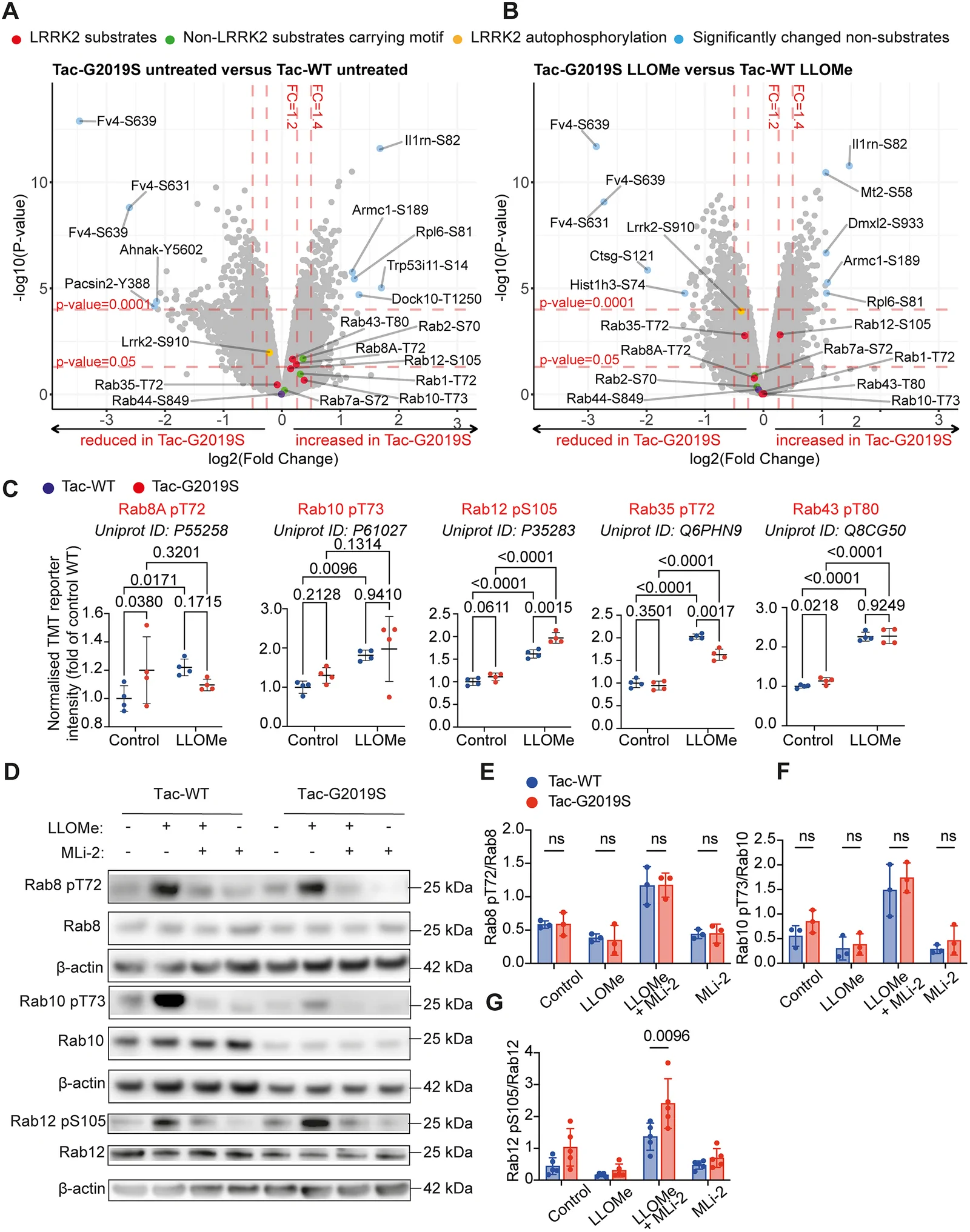
**LRRK2 G2019S selectively rewires Rab phosphorylation following lysosomal damage.** (A,B) BMDMs were treated with 1 mM LLOMe for 30 min and cells were analysed by TMTpro 16-plex mass spectrometry. Volcano plots of the phosphoproteome in the untreated (A) and LLOMe-treated (B) conditions showing the difference in phosphorylation levels between Tac-WT and Tac-G2019S BMDMs. Each point represents one phosphorylation site of a protein. The *x*-axis shows the log_2_-transformed fold change (FC), and the *y*-axis shows the significance by −log_10_-transformed *P*-value, obtained by linear models for microarray data. FC>1.2 and *P*<0.05 were deemed significant. (C) Scatterplots showing the normalised TMT reporter intensity data obtained by mass spectrometry and *P*-values for individual Rabs sites that were phosphorylated by LRRK2 as identified in the previous experiment. CONSTANd-normalised and log_2_-transformed raw TMT reporter intensities were back-transformed (2^x^) and expressed relative to the mean of the control WT group (set at 1). *n*=4 biological replicates per condition. *P*-values were calculated by linear models for microarray data (limma) statistics on the normalised log_2_-transformed data. Error bars represent s.d.. (D) Western blot analysis of Rab8 pT72, Rab8, Rab10 pT73, Rab10, Rab12 pS105, Rab12 and β-actin in Tac-WT and Tac-G2019S macrophages in untreated, LLOMe, LLOMe+MLi-2 and MLi-2 conditions. (E–G) Rab8 pT72, Rab10 pT73 and Rab12 pS105 band intensities were quantified by densitometry and normalised to Rab8, Rab10 and Rab12 band intensities, respectively. Data are mean±s.d. from (E,F) three and (G) five independent experiments. Two-way ANOVA followed by Šidák's multiple comparisons test. ns, not significant.

To determine how the pathogenic LRRK2 G2019S variant affects substrate phosphorylation in macrophages, we performed phosphoproteomic analysis in BMDMs expressing endogenous levels of wild-type LRRK2 (Tac-WT) or LRRK2 G2019S (Tac-G2019S). We identified 32,703 phosphosites. Significant changes (|log_2_FC|>1.2 and *P*<0.05) were observed in 3732 sites (2108 reduced and 1624 increased in Tac-G2019S) in the control group (Fig. 2A) and 4390 sites (2193 reduced and 2197 increased in Tac-G2019S) in the LLOMe-treated group (Fig. 2B). Tac-WT and Tac-G2019S macrophages showed similar levels of macrophage surface marker expression by flow cytometry, and no differences in lysosomal content, proteolytic activity or LRRK2 expression (Fig. S3A–I).

In contrast to the expectation that LRRK2 G2019S broadly enhances kinase activity, we did not observe widespread increases in Rab phosphorylation in the basal state (Fig. 2A,C). Instead, lysosomal damage induced phosphorylation of multiple Rab GTPases in both Tac-WT and Tac-G2019S macrophages, consistent with activation of LRRK2 signalling in this context (Fig. 2A–C). However, direct comparison between genotypes revealed a selective alteration in substrate phosphorylation. Specifically, Rab12 phosphorylation was significantly increased in Tac-G2019S macrophages, whereas Rab35 phosphorylation was reduced, and other Rab substrates showed no consistent differences (Fig. 2B,C). Our proteomic analysis identified 6720 proteins. Significant changes (|log_2_FC|>1.2 and *P*<0.05) were observed in 235 proteins (136 reduced and 99 increased in Tac-G2019S) in the control group (Fig. S4A) and 232 proteins (126 reduced and 106 increased in Tac-G2019S) in the LLOMe-treated group (Fig. S4B). Total protein levels of these Rab GTPases were unchanged, indicating that these effects reflect selective modulation of phosphorylation rather than altered expression (Fig. S4A,B).

These findings were confirmed by western blot analysis, which showed increased Rab12 phosphorylation in Tac-G2019S macrophages following lysosomal damage, and this effect was reversed by LRRK2 kinase inhibition (Fig. 2D–G). In contrast, phosphorylation of Rab8 and Rab10 was not significantly altered between genotypes (Fig. 2D–G).

In addition to Rab GTPases, we identified changes in the phosphorylation of a limited number of non-Rab proteins following lysosomal damage in Tac-G2019S macrophages. However, these changes were modest and not associated with consistent alterations in protein abundance, suggesting that they are unlikely to represent major direct substrates of LRRK2 in this context (Fig. 2A,B; Fig. S4C–E). Taken together, these data indicate that the pathogenic G2019S variant alters the specificity of LRRK2 signalling rather than its overall activity, resulting in selective redistribution of phosphorylation across Rab substrates following lysosomal damage. This selective rewiring of Rab phosphorylation suggests that pathogenic LRRK2 differentially regulates downstream membrane trafficking pathways.

### LRRK2 G2019S increases susceptibility to lysosomal damage-induced cell death independently of kinase activity

**Fig. 3.**
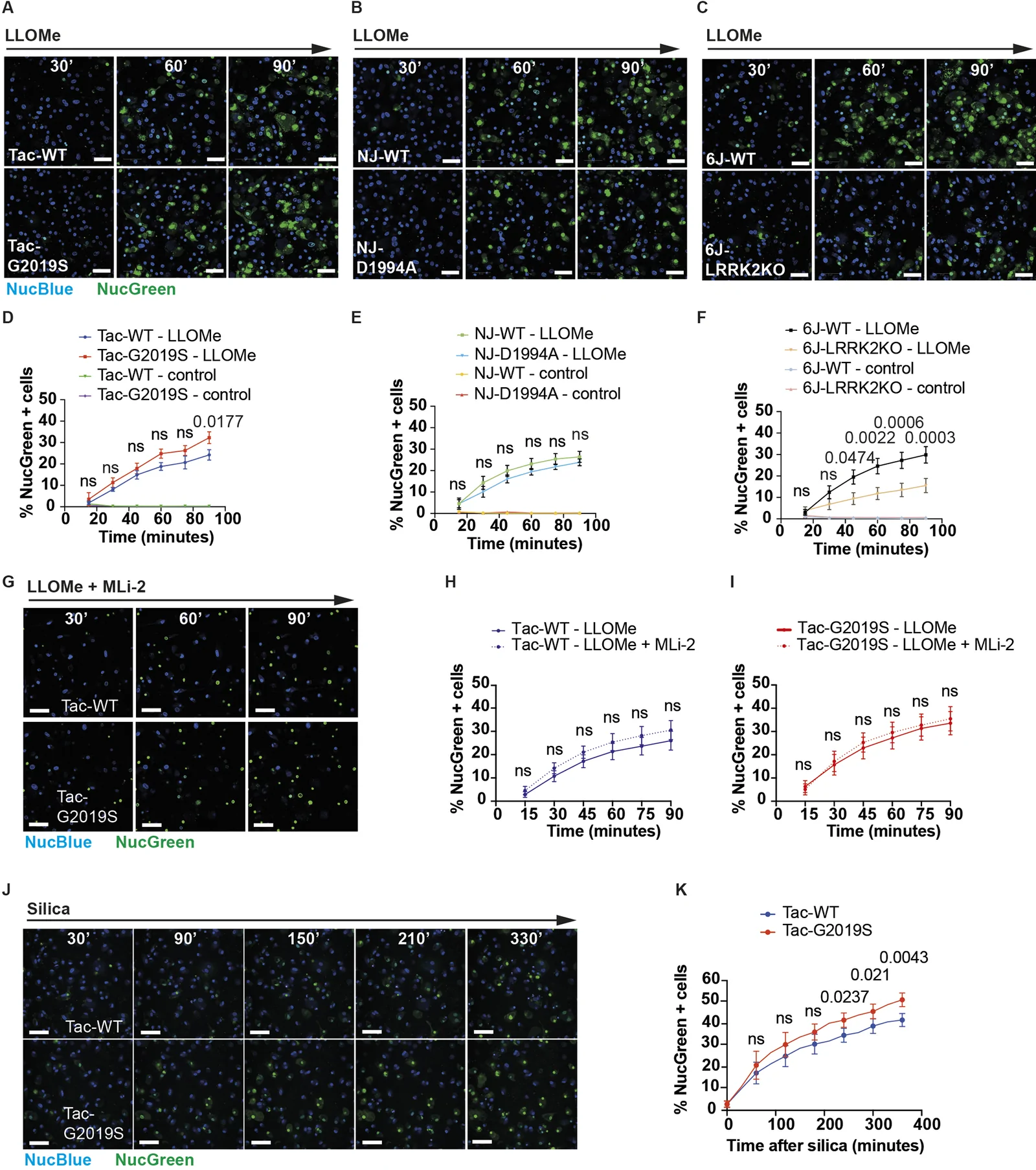
**LRRK2 G2019S increases susceptibility to lysosomal damage-induced cell death independently of kinase activity in mouse primary macrophages.** (A–C) Snapshot of live BMDMs treated with 1 mM LLOMe and imaged every 15 min. Nuclear staining for live (blue) and dead (green) cells was performed. Scale bars: 50 μm. (D–F) Quantitative analysis of the percentage of dead cells at each timepoint. Two-way ANOVA (mixed-effects analysis) followed by Bonferroni's multiple comparisons test. Data shown are the mean±s.e.m. from (D) six and (E,F) three independent experiments. (G) Snapshot of live BMDMs treated with 1 mM LLOMe and 100 nM MLi-2 and imaged every 15 min. Nuclear staining for live (blue) and dead (green) cells was performed. Scale bars: 50 μm. (H,I) Quantitative analysis of the percentage of dead cells at each timepoint. Two-way ANOVA (mixed-effects analysis) followed by Bonferroni's multiple comparisons test. Data shown are the mean±s.e.m. from three independent experiments. (J) Snapshot of live BMDMs treated with 300 μg/ml silica and imaged every 30 min. Nuclear staining for live (blue) and dead (green) cells was performed. Scale bars: 50 μm. (K) Quantitative analysis of the percentage of dead cells at each timepoint. Data shown are the mean±s.d. from three independent experiments. Two-way ANOVA followed by Šidák's multiple comparisons test. ns, not significant.

We next asked whether lysosomal damage alters macrophage fate. To address this, we quantified cell death in Tac-WT and Tac-G2019S macrophages using live-cell imaging. Following LLOMe-induced lysosomal damage, Tac-G2019S macrophages exhibited significantly increased cell death compared to Tac-WT cells (Fig. 3A,D). This difference became more pronounced over time and was consistently observed across experiments, indicating that G2019S lowers the threshold for lysosomal damage-induced cell death. Importantly, this phenotype was not attributable to differences in the extent of initial lysosomal damage, as both genotypes exhibited comparable responses immediately following damage induction (Fig. 3A,D).

To determine whether this increased susceptibility depends on LRRK2 kinase activity, we assessed cell death in macrophages expressing a kinase-dead LRRK2 mutant (NJ-D1994A) and in LRRK2 knockout cells (6J-LRRK2KO), alongside their respective wild-type controls. Macrophages expressing kinase-dead LRRK2 (D1994A) showed no increase in cell death relative to wild-type cells (Fig. 3B,E), indicating that loss of kinase activity alone is not sufficient to account for the G2019S phenotype. Similarly, LRRK2 knockout macrophages did not display increased susceptibility to lysosomal damage-induced cell death and instead showed reduced levels of cell death compared to wild-type controls (Fig. 3C,F), further supporting the conclusion that the G2019S phenotype is not simply due to altered kinase activity. Consistent with this, pharmacological inhibition of LRRK2 kinase activity using MLi-2 did not rescue the increased cell death observed in Tac-G2019S macrophages (Fig. 3G–I). To confirm that this phenotype is not restricted to LLOMe-induced damage, we assessed cell death following silica-induced lysosomal injury (Chen et al., 2026). LRRK2 G2019S macrophages again exhibited increased cell death compared to wild-type controls (Fig. 3J,K), indicating that enhanced susceptibility to lysosomal stress is a general feature of the G2019S variant. Taken together, these findings demonstrate that LRRK2 G2019S promotes cell death following lysosomal damage through a mechanism that is independent of kinase activity, suggesting a distinct noncanonical function of LRRK2 in regulating cell survival.

### LRRK2 G2019S promotes apoptosis following lysosomal damage independently of kinase activity

**Fig. 4.**
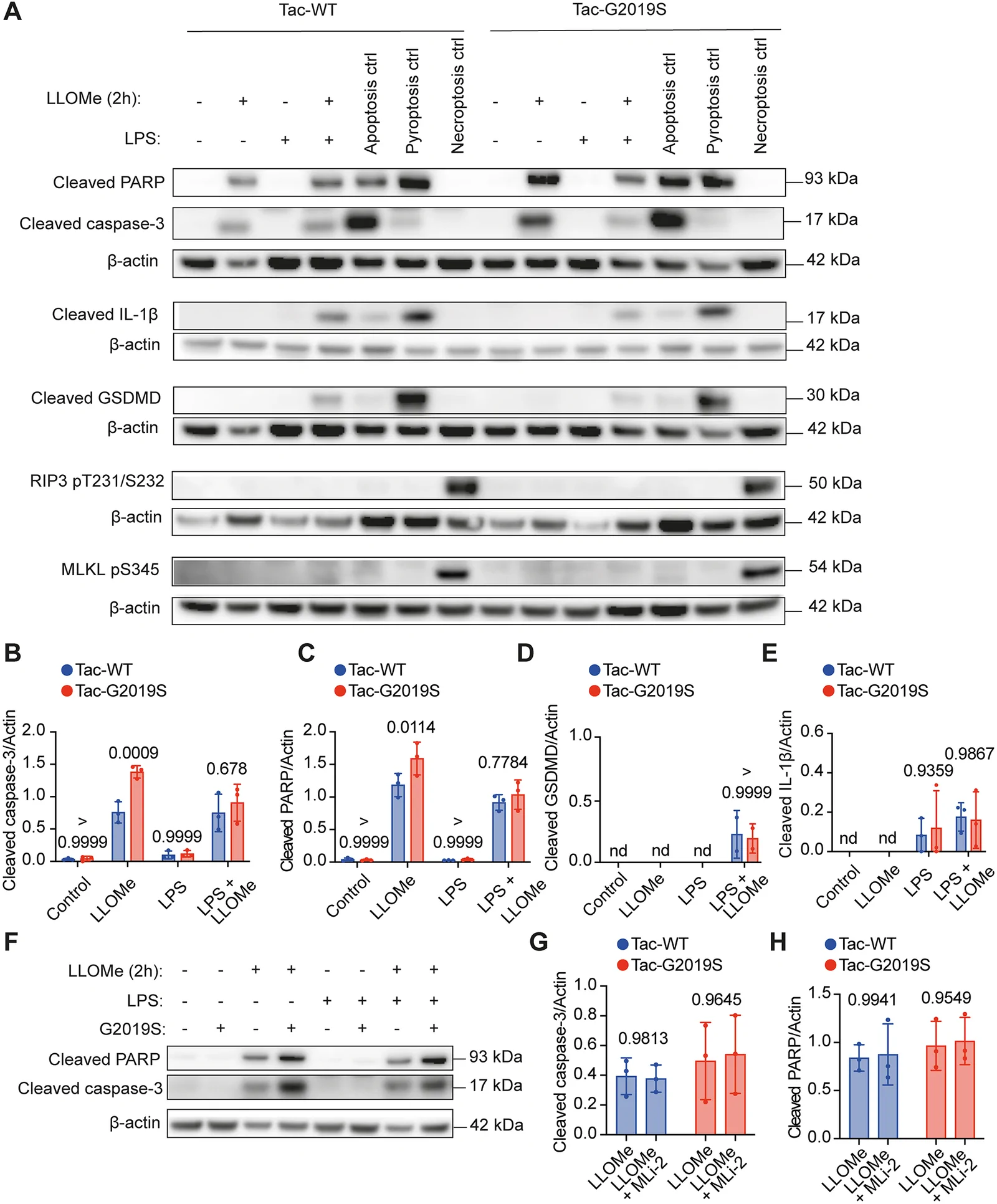
**LRRK2 G2019S promotes apoptosis following lysosomal damage independently of kinase activity.** (A) Tac-WT and Tac-G2019S BMDMs were pre-treated with 250 ng/ml LPS or vehicle for 3 h, followed by 1 mM LLOMe for 2 h. For induction of apoptosis, BMDMs were treated with 100 ng/ml TNF-α and 125 nM 5Z-7-oxozeaenol for 7 h. For induction of necroptosis, BMDMs were treated with 100 ng/ml TNF-α, 125 nM 5Z-7-oxozeaenol and 10 μM Z-VAD-FMK for 7 h. For induction of pyroptosis, BMDMs were pre-treated with 250 ng/ml LPS for 3 h followed, by 20 μM nigericin for 3 h. Western blots for cleaved PARP, cleaved caspase-3, cleaved IL-1β, cleaved gasdermin D (GSDMD), phospho-RIP3, phospho-MLKL and β-actin are shown. The β-actin blot shown for cleaved PARP, cleaved caspase-3 and cleaved GSDMD represents a shared control for these blots, which were all performed using the same membrane. (B–E) Protein levels were quantified by densitometry and normalised to β-actin. Data represent the mean±s.d. from three independent experiments. Two-way ANOVA followed by Šidák's multiple comparisons test. (F) BMDMs were pre-treated with 100 nM MLi-2, followed by 1 mM LLOMe for 2 h. Western blots for cleaved PARP, cleaved caspase-3 and β-actin are shown. (G,H) Protein levels were quantified by densitometry and normalised to β-actin. Data represent the mean±s.d. from three independent experiments. Two-way ANOVA followed by Šidák's multiple comparisons test. ctrl, control; nd, not detected.

To define the mode of cell death induced by lysosomal damage in Tac-G2019S macrophages, we assessed markers of apoptotic, pyroptotic and necroptotic cell death pathways. Following lysosomal damage, we observed cleavage of PARP1 (hereafter PARP) and caspase-3, indicating activation of apoptosis (Fig. 4A–C). Notably, levels of cleaved PARP and caspase-3 increased in Tac-G2019S macrophages compared to Tac-WT cells, consistent with the enhanced susceptibility to cell death observed in these cells (Fig. 4A–C). Markers of necroptosis [RIP3 (also known as RIPK3) phospho(p)-T231/S232 and MLKL pS346] were not detected following lysosomal damage (Fig. 4A) and, although pyroptosis [detected by cleaved IL-1β (IL1B) and cleaved gasdermin D (GSDMD)] was induced under lipopolysaccharide (LPS)-priming conditions, its activation was comparable between Tac-WT and Tac-G2019S macrophages (Fig. 4A–E). These data suggest that the enhanced cell death observed in Tac-G2019S macrophages is not driven by necroptotic or pyroptotic pathways.

To determine whether this phenotype depends on LRRK2 kinase activity, we inhibited LRRK2 using MLi-2. In line with our previous findings, kinase inhibition did not reduce PARP or caspase-3 cleavage in Tac-G2019S macrophages, indicating that the increase in apoptosis occurs independently of LRRK2 kinase activity (Fig. 4F–H). Together, these results demonstrate that LRRK2 G2019S selectively promotes apoptosis following lysosomal damage through a mechanism independent of kinase activity, indicating a distinct role for LRRK2 in regulating macrophage cell survival.

### LRRK2 G2019S increases susceptibility to lysosomal damage-induced cell death independently of kinase activity in human iPSC-derived macrophages

To validate these findings in a human system, we examined macrophages derived from iPSCs from patients with PD carrying the G2019S variant and isogenic controls (Bernard et al., 2020; van Wilgenburg et al., 2013). We performed CRISPR/Cas9 gene editing in iPSCs from a patient with the LRRK2 G2019S variant to generate isogenic iPSCs (see Materials and Methods and Fig. S5). Isogenic and G2019S iPSCs showed similar differentiation into iPSC-derived macrophages (iPSDMs) by expression of macrophage markers and showed no differences in LysoTracker intensity, proteolytic activity or changes in LRRK2 expression (Fig. S6A–G). Furthermore, we confirmed that isogenic and G2019S iPSDMs showed increased LRRK2 kinase activity after lysosomal damage, as measured by phosphorylation of Rab8, Rab10 and Rab12, and this phosphorylation was inhibited by the LRRK2 kinase inhibitor MLi-2 (Fig. S6H–K). There was a trend for increased phosphorylation of Rab12 S106 in G2019S iPSDMs compared to that in isogenic controls (Fig. S6H,K), in agreement with the results from mouse macrophages.

**Fig. 5.**
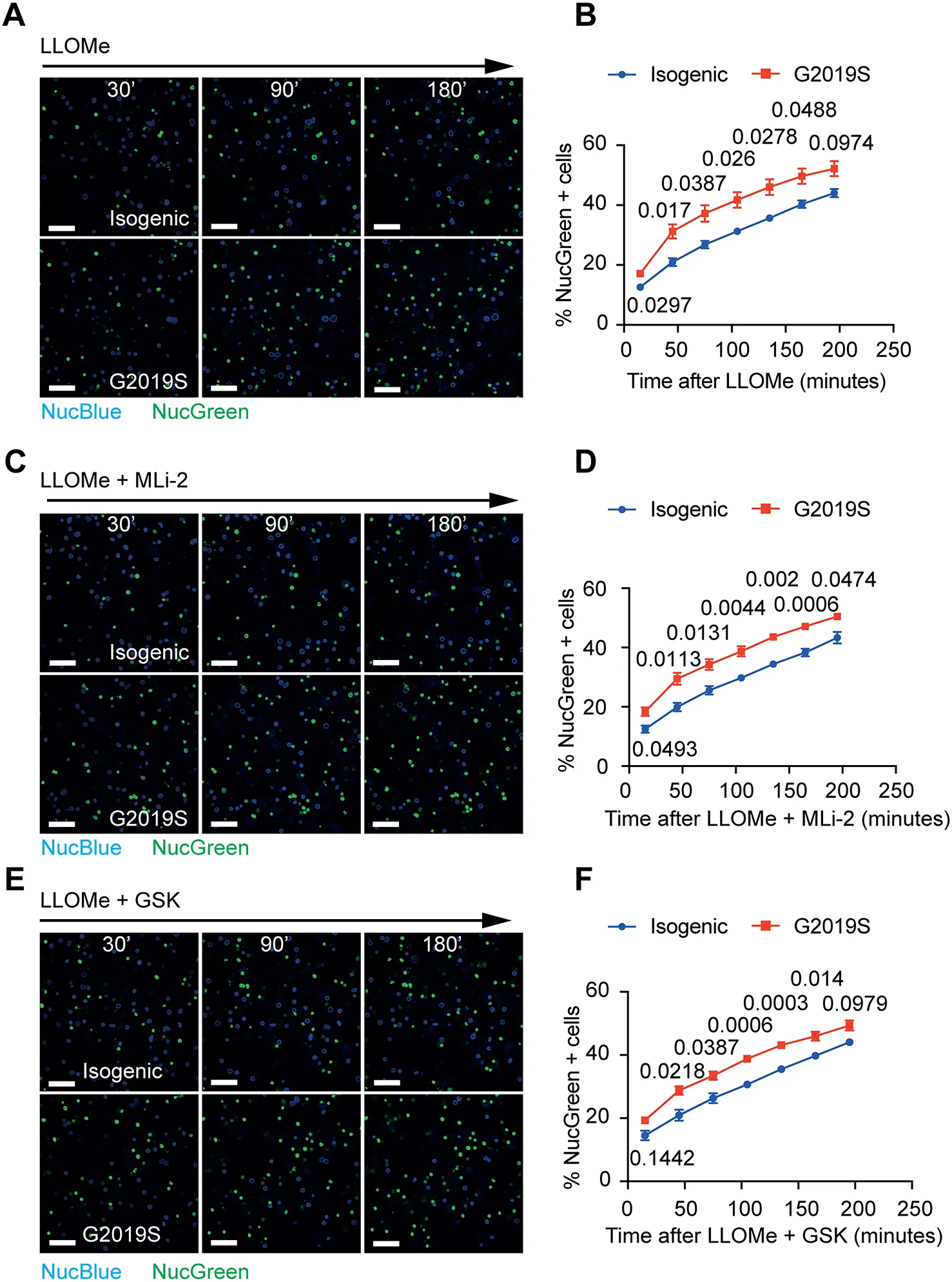
**LRRK2 G2019S increases susceptibility to lysosomal damage-induced cell death independently of kinase activity in patient-derived iPSDMs.** (A,C,E) Snapshot of live induced pluripotent stem cell-derived macrophages (iPSDMs) (clone ID: CDI00002173 and isogenic) treated with 1 mM LLOMe and imaged every 15 min. Cells were pre-treated with the LRRK2 inhibitors MLi-2 or GSK2578215A (GSK) for 2 h where indicated. Nuclear staining for live (blue) and dead (green) cells was performed. Scale bars: 50 μm. (B,D,F) Quantitative analysis of the percentage of dead cells at each timepoint. Data shown are the mean±s.d. from three independent experiments. Two-way ANOVA followed by Šidák's multiple comparisons test.

Consistent with the data from mouse macrophages, LRRK2 G2019S human macrophages showed increased susceptibility to cell death following lysosomal damage (Fig. 5A,B). This effect was independent of kinase activity, as treatment with the LRRK2 inhibitors MLi-2 and GSK2578215A (GSK) did not alter cell death levels (Fig. 5C–F). This finding was reproduced in a second independent patient-derived iPSC clone (clone ID: CDI00008375), confirming that enhanced susceptibility to lysosomal damage-induced cell death is a consistent feature of LRRK2 G2019S macrophages (Fig. S7). Together, these results demonstrate that LRRK2 G2019S enhances susceptibility to lysosomal damage-induced cell death through a mechanism that is independent of kinase activity, indicating a distinct non-catalytic role of LRRK2 in regulating macrophage survival.

## DISCUSSION

In this study, we identify lysosomal damage as a critical context in which LRRK2 regulates macrophage function and demonstrate that the pathogenic G2019S variant disrupts this response. Rather than inducing global kinase hyperactivation, LRRK2 G2019S selectively redistributes Rab GTPase phosphorylation following lysosomal damage.

A central observation of this study is that LRRK2 G2019S does not broadly enhance Rab phosphorylation but instead selectively alters substrate specificity, characterised by increased Rab12 phosphorylation and reduced Rab35 phosphorylation. This finding challenges the prevailing view that G2019S primarily acts through global kinase hyperactivation (Greggio et al., 2006; Henry et al., 2015; Smith et al., 2006; Yadavalli and Ferguson, 2023) and instead suggests that pathogenic LRRK2 reshapes downstream signalling outputs. Given the established roles of Rab GTPases in membrane trafficking (Gutierrez, 2013; Prashar et al., 2017), this selective rewiring is likely to have functional consequences for pathways that coordinate lysosomal dynamics. Our data indicate that pathogenic LRRK2 changes substrate channelling rather than uniformly increasing substrate phosphorylation, consistent with the idea that different Rab GTPases (e.g. Rab12 or Rab35) control distinct membrane trafficking pathways. The selective pRab12 increase and pRab35 decrease we observed offer two candidate routes to inefficient recovery after lysosomal damage without implicating global kinase hyperactivation. Further work will elucidate how differential Rab GTPase phosphorylation is implicated in lysosomal damage.

Crucially, our data indicate that LRRK2 G2019S increases susceptibility to lysosomal damage-induced cell death through a mechanism that is independent of kinase activity. This phenotype is observed in both mouse and human macrophages and is associated with increased apoptotic signalling, as indicated by elevated caspase-3 and PARP cleavage. The kinase independence of this effect indicates that LRRK2 exerts non-catalytic functions in regulating cell survival, consistent with emerging evidence that LRRK2 can act as a scaffold or regulator of protein interactions (Price et al., 2018; Zhao et al., 2023). These findings suggest that pathogenic variants alter LRRK2 conformation or interaction networks, thereby modulating downstream signalling independently of kinase activity. The macrophage phenotypes we uncover intersect with PD-relevant processes. In macrophage-lineage cells, α-synuclein fibrils can impose lysosomal stress that engages the LRRK2–Rab10 axis and promotes α-synuclein release, supporting a feed-forward cycle of proteopathic spread (Abe et al., 2024). In this context, a LRRK2 G2019S-driven failure to repair lysosomal membranes coupled with increased apoptosis might lower the threshold for inflammatory damage, cell loss or maladaptive vesicle trafficking, providing an immune-mediated model of LRRK2 pathobiology.

It is important to consider gene dosage when interpreting the magnitude of phenotypes observed across model systems. In our study, mouse BMDMs carry the G2019S variant in a homozygous context (Matikainen-Ankney et al., 2016), whereas patient-derived iPSC macrophages are heterozygous, reflecting the genetic state found in individuals with PD. As a result, the effects observed in human macrophages are expected to be more modest than those seen in mouse models. Indeed, we detected a consistent, modest increase in susceptibility to lysosomal damage-induced cell death in G2019S iPSC-derived macrophages, indicating that even partial expression of the mutant allele is sufficient to alter cellular responses to stress. These findings support the physiological relevance of our observations while highlighting the importance of gene dosage in shaping LRRK2-dependent phenotypes.

These findings have broader implications for understanding LRRK2 function in disease. The relatively low penetrance of the G2019S variant (Trinh et al., 2022) suggests that its effects are context dependent and become apparent under conditions of cellular stress. Our data support this idea by demonstrating that lysosomal damage reveals functional defects that are not evident under basal conditions. In immune cells, which are frequently exposed to lysosomal stress during host defence and inflammation, such defects might contribute to dysregulated cell death and altered immune responses. More broadly, the identification of kinase-independent functions of LRRK2 highlights potential limitations of therapeutic strategies that focus solely on kinase inhibition, and suggests that targeting LRRK2 conformation or protein interactions is required to fully modulate its pathogenic effects.

Although our phosphoproteomic analysis robustly identifies selective altered phosphorylation in G2019S macrophages, the link between increased Rab12 phosphorylation, decreased Rab35 phosphorylation and cell death requires further studies and new tools. Loss-of-function or phospho-mutant Rab12 and Rab35 rescue experiments are a priority for future work. Notably, a complementary study found that in LRRK2 G2019S macrophages, iron overload triggers a block in NCOA4 degradation and a pronounced accumulation of phosphorylated Rab8 at the plasma membrane, driving ferroptotic cell death; as in our system, this phenotype was resistant to the type I kinase inhibitor MLi-2 but was reversed by type II LRRK2 inhibitors (Goldman et al., 2026). Finally, our cell death and apoptosis data point to a kinase-independent role for LRRK2; future work should delineate whether conformational and/or scaffolding mechanisms of LRRK2 G2019S underlie macrophage vulnerability.

In summary, these data show that LRRK2 G2019S reshapes macrophage responses to lysosomal damage by selectively rewiring Rab GTPase signalling and triggering cell death. These findings provide a framework for understanding how pathogenic LRRK2 variants alter immune cell function and reveal new avenues for therapeutic intervention in PD.

## MATERIALS AND METHODS

### Animals

Depending on the mouse strain, breeding pairs, embryos or sperm were purchased from Taconic Biosciences or Jackson laboratories. The strains used were C57BL/6NTac (Tac-WT), C57BL/6-Lrrk2^tm4.1Arte^ (Tac-G2019S), C57BL/6NJ (NJ-WT) and B6(SJL)-Lrrk2^tm4.1Mjff/J^ (NJ-D1994A). Mice were re-derived, bred in-house and maintained under specific pathogen-free conditions at the Francis Crick Institute (Panagiotakopoulou et al., 2020). Animal studies and breeding were approved by the Francis Crick Institute ethical committee and performed under UK Home Office project license P4D8F6075. All animal studies were ethically reviewed and carried out in accordance with Animals (Scientific Procedures) Act 1986.

### Culture of murine primary BMDMs

Bone marrow was isolated by flushing the femur and tibia of 6- to 10-week-old female mice with ice-cold PBS as described previously (Schnettger and Gutierrez, 2017). Bone marrow cells were differentiated in RPMI 1640 medium (72400-021, Gibco) containing 10% fetal bovine serum (FBS; 10270-106, Gibco) and 50 ng/ml GM-CSF (576306, BioLegend) for 7 days. For immunofluorescence and live-cell snapshot imaging, 6×10^4^ cells were seeded per well in 96-well olefin-bottomed plates (6055302, Revvity). For western Blotting, 5×10^5^ cells were seeded per well in 12-well plates (353043, Falcon) or 1×10^6^ cells were seeded per well in six-well plates (353046, Falcon).

### Gene editing to generate isogenic iPSCs

Isogenic control iPSCs were obtained using the CRISPR/Cas9 system. We designed a two-step approach. Briefly, nucleofection using the Amaxa 4D-Nucleofector (V4XP-3024, Lonza) was performed in the LRRK2 G2019S iPSCs (clone IDs: CDI00002173 and CDI00008375) obtained from the Michael J. Fox Foundation (MJFF) Parkinson's Progression Markers Initiative (PPMI) resource. For each nucleofection, 1×10^6^ iPSCs were resuspended in 100 μl of P3 buffer (V4XP-3024, Lonza) containing 20 μg Alt-R S.p. Cas9 Nuclease V3 (1081059, IDT) mixed with 16 μg targeting synthetic chemically modified single guide RNA (sgRNA; from Synthego) (Table S1, LRRK2-1step), as previously described (Skarnes et al., 2019). At this point, we screened and selected only the clones containing an indel in the allele harbouring the G2019S variant by using the ICE platform from Synthego (https://ice.synthego.com/#/). Then, we designed a new sgRNA targeting the allele containing the indel (Table S1, LRRK2-2step) and performed nucleofection, for which 1×10^6^ cells of the selected clone were resuspended in 100 μl of P3 buffer containing 20 μg Alt-R S.p. Cas9 Nuclease V3, mixed with a total of 16 μg of the sgRNAs and 200 pmol of single-stranded oligodeoxynucleotides (Table S1), as described before (Skarnes et al., 2019). We screened the clones by Sanger sequencing and selected clones with the wild-type sequence (Table S2). As a result, we obtained multiple independent clones, and we proceeded to choose one clone for use in future experiments. The clones used in this study are LRRK2-G2019S and isogenic control iPSCs [clone IDs: CDI00002173 and 0043052321 (isogenic); CD10008375 and 0043052324 (isogenic)]. Sanger sequencing results are shown in Fig. S5. The edited locus was amplified by PCR and the amplicon was Sanger sequenced (primers listed in Table S1). The same primers were used for routine genotyping of the clones. Confirmed corrected clones, exhibiting the reversal of the G2019S variant, were subjected to further analyses, including testing for mycoplasma contamination, assessment of pluripotency and karyotyping. All clones were genotyped and banked for long-term storage.

### Culture of iPSCs and iPSDMs

LRRK2 G2019S iPSCs (clone IDs: CDI00002173 and CDI00008375) were obtained from the MJFF PPMI resource. iPSCs were maintained feeder-free on vitronectin (A14700, Life Technologies) in Essential 8 medium (A1517001, Life Technologies) at 37°C and 5% CO₂, with daily medium changes. We found that these lines showed poor viability on thawing when cryopreserved in conventional DMSO-based freezing medium. Cells were therefore cryopreserved and thawed using the PSC Cryopreservation Kit (A2644601, Thermo Fisher Scientific) according to the manufacturer's instructions, which markedly improved post-thaw recovery. Cells were thawed in the presence of 10 µM Y-27632 ROCK inhibitor (Tocris Bioscience) for the first 24 h. iPSCs were used to generate iPSDMs following a previously reported protocol using embryoid bodies (Bernard et al., 2020; Shapouri-Moghaddam et al., 2018; van Wilgenburg et al., 2013). Briefly, embryoid bodies were fed daily with two partial medium changes (replacing half the medium with fresh medium) comprising E8 medium (A1517001, Life Technologies), 50 ng/ml BMP4 (120-05, Peprotech), 50 ng/ml VEGF (100-20, Peprotech) and 20 ng/ml SCF (300-07, Peprotech). On day 4, embryoid bodies were transferred to T225 cm^2^ flasks for macrophage differentiation using factory medium consisting of X-VIVO 15 (LZBE02-061Q, Lonza), 2 mM GlutaMAX (35050061, Life Technologies), 50 μM β-mercaptoethanol (31350010, Life Technologies), 100 ng/ml M-CSF (300-25, Peprotech) and 25 ng/ml IL-3 (200-03, Peprotech). 20 ml fresh factory medium was added once per week. After 4–5 weeks, macrophage precursors were collected from the supernatant. These precursors were differentiated into macrophages in X-VIVO 15 containing 2 mM GlutaMAX and 100 ng/ml M-CSF for 7 days. Macrophages were detached using Versene (15040066, Life Technologies), centrifuged at 300 ***g*** and plated for experiments.

### Antibodies

The primary antibodies used for western blotting, immunofluorescence and flow cytometry are listed in Table S3. For western blotting, the secondary antibody used was anti-rabbit-HRP conjugate (W410B, Promega, 1:5000 dilution). For immunofluorescence, the secondary antibodies used were Alexa Fluor 488 goat anti-rabbit IgG (H+L) (A11034, Life Technologies) and Cy3 goat anti-rabbit IgG (H+L) (A10520, Life Technologies) (1:800 dilution).

### LLOMe, MLi-2, GSK2578215A and silica treatment

LLOMe (4000725, Bachem) was prepared in methanol at a stock concentration of 333 mM and frozen at −20°C. Cells were treated with LLOMe at a concentration of 1 mM by dilution in RPMI (BMDMs) or X-VIVO 15 (iPSDM). 0.3% methanol in RPMI or X-VIVO 15 was used in all control samples. Cells were pre-treated with MLi-2 (5756/10, Tocris) at 100 nM or GSK2578215A (ab254316, Abcam) at 0.1 μM for 2 h and all subsequent treatments were performed in the presence of MLi-2 or GSK2578215A. Stock crystalline silica (MIN-U-SIL-15, US Silica) was prepared at 30 mg/ml by dilution in PBS immediately prior to use. A working solution of 300 μg/ml crystalline silica was prepared in RPMI for subsequent treatments.

### Phosphoproteomics

#### Sample preparation and processing

For LLOMe treatment, 5×10^5^ cells/ml were plated in three 50 mm non-treated tissue culture dishes (122-17, Thermo Fisher Scientific). For controls, 6×10^5^ cells/ml were plated in a 90 mm Petri dish (101R20, Thermo Fisher Scientific). Cells were treated with LLOMe for 30 min at a concentration of 1 mM by dilution in RPMI medium. Following treatment, cells were washed once in ice-cold PBS and then detached with ice-cold PBS containing sigma phosphatase inhibitor cocktail 3 (539134, Merck), cOmplete protease inhibitor tablets (11873580001, Merck), Pierce phosphatase inhibitor mini tablets (A32957, Thermo Fisher Scientific), 500 nM okadaic acid (A4540, Stratech) and 1 mM EDTA (11568896, Invitrogen). A pellet was collected by centrifugation (350 ***g*** for 5 min) and the pellet was flash-frozen using dry ice and isopropanol together as a slurry bath. Four replicates were collected per condition (control and LLOMe treatments) and genotype [NJ-WT and NJ-D1994A (set 1), and Tac-WT and Tac-G2019S (set 2)] for a total of 16 samples per set. Pellets were lysed in urea lysis buffer [8 M urea, 50 mM HEPES pH 8.2, 10 mM glycerol-2-phosphate, 50 mM NaF, 5 mM sodium pyrophosphate, 1 mM EDTA, 1 mM EGTA, 1 mM sodium vanadate, 1 mM dithiothreitol (DTT), 2× cOmplete protease inhibitor cocktail (Merck), 1× phosphatase inhibitor cocktail 3 (Sigma-Aldrich), 500 nM okadaic acid, 1 μM microcystin-LR (Sigma-Aldrich)]. After the protein concentration was measured using the Pierce BCA protein assay kit (23227, Thermo Fisher Scientific), an equal amount (between 165–200 μg) of each sample was used for TMTpro multiplexed quantitative proteomics. Each sample was reduced with 10 mM DTT for 1 h at 56°C and alkylated with 20 mM iodoacetamide for 30 min in the dark at room temperature. The reaction was quenched with 20 mM DTT, then diluted to <2 M urea with 50 mM HEPES pH 8.5. Each resuspended sample was digested with 3.75 μg LysC (lysyl endopeptidase, 125-05061, FUJIFILM Wako Chemicals) and 12.5 μg trypsin [mass spectrometry (MS) grade, 90058, Thermo Fisher Scientific] at 37°C shaking overnight. Each sample was cleaned up using a BioPureSPN Macro spin column (Proto 300 C18, HMM S18V, Nest Group) and dried by vacuum centrifugation. Samples were then TMT-labelled for 1 h at room temperature using a separate TMTpro 16-plex isobaric label reagent set (labels aliquoted to 0.8 mg per tag, A44520, lot VE299607, Thermo Fisher Scientific) for each set of samples and following the manufacturer's instructions. A small aliquot of each sample was collected for labelling efficiency and mixing-accuracy quality checks by liquid chromatography tandem mass spec (LC-MS/MS) using an Orbitrap Eclipse Tribrid mass spectrometer (Thermo Fisher Scientific) and a 60 min higher-energy collisional dissociation (HCD) MS2 fragmentation method. The rest of the sample was stored at −80°C until quality check results. A labelling efficiency of higher than 99% was obtained for each reaction and a mixing accuracy with lower than 1.5× difference between samples with lowest and highest summed intensity was obtained. Samples were defrosted, quenched with hydroxylamine to a final concentration of 0.2% for 15 min at room temperature and pooled together (sets 1 and 2 were pooled separately). The combined mixture for each set was partially dried by vacuum centrifugation and acidified to pH 2.0, followed by sample clean-up using a Sep Pak C18 1cc Vac cartridge with 100 mg bed volume (Waters, WAT023590). The final mixing check was performed by LC-MS/MS with a 240 min HCD MS2 fragmentation method. The peptide mixture was then subjected to high-select sequential enrichment of metal oxide affinity chromatography (SMOAC) to capture phosphopeptides. It was first passed through a high-select TiO_2_ phospho-enrichment column (Thermo Fisher Scientific, A32993) following the manufacturer's protocol. Flow-through and wash fractions were combined, dried and subsequently used for Fe-NTA phospho-enrichment (Thermo Fisher Scientific, A32992). One tenth of the combined flow-through and wash fractions from this enrichment was used for proteome analysis. The eluates from SMOAC were dried by vacuum centrifugation, solubilised and pooled together. Both proteome and phosphoproteome samples were subjected to high-pH reversed-phase fractionation by solubilising in 2% trifluoroacetic acid (TFA) and using elution solutions for ‘Thermo Scientific TMT-labelled peptides’ (proteome) or ‘unlabelled, native peptides’ (phosphoproteome) as outlined in the manufacturer's protocol (Thermo Fisher Scientific, 84868). Fractions were then dried by vacuum centrifugation.

#### Data acquisition

Dried samples were resolubilised in 0.1% TFA prior to being analysed by online nanoflow LC-MS/MS using an Orbitrap Eclipse Tribrid mass spectrometer (Thermo Fisher Scientific) coupled to a UltiMate 3000 RSLCnano system (Thermo Fisher Scientific). Samples were loaded via the autosampler and concentrated onto a trap column (75 μm×2 cm, Acclaim PepMap 100 C18, 3 μm particle size, 100 Å pore size; PN 164535, Thermo Fisher Scientific) using loading buffer [2% (v/v) acetonitrile, 0.05% (v/v) TFA, 97.95% water] at a flow rate of 7 μl/min for 6 min in the column oven at 40°C. Peptides were then gradient eluted onto an EASY-Spray analytical column (75 μm×50 cm, PepMap RSLC C18, 2 μm particle size, 100 Å pore size; PN ES903, Thermo Fisher Scientific) at a flow rate of 300 nl/min, with the column temperature held at 40°C, and a typical spray voltage of 2.1 kV using the EASY-Spray Source (Thermo Fisher Scientific). Gradient elution buffers were: A, 0.1% (v/v) formic acid, 5% (v/v) DMSO, 94.9% (v/v) water; and B, 0.1% (v/v) formic acid, 5% (v/v) DMSO, 20% (v/v) water and 74.9% (v/v) acetonitrile. The gradient elution profile was 2% B to 8% B for 5.5 min, 8% B to 40% B for 147 min, and 40% B to 95% B for 1 min. For all methods, Xcalibur software (Thermo Fisher Scientific, RRID:SCR_014593) was used to control the data acquisition on the Orbitrap Eclipse Tribrid Mass Spectrometer. The instrument was run in data-dependent acquisition mode with the most abundant peptides selected for MS/MS using a 3 s cycle time. In all cases, the typical capillary temperature used was 300°C, the radiofrequency ion lens was set to 30%, the Orbitrap resolution for the MS1 scan was 120,000, the maximum injection time was 50 ms, and the automatic gain control (AGC) target was 100%. The proteome fractions were analysed using the 180 min MS2 method with the HCD fragmentation (method 1), and a 180 min real-time search (Zabetian et al., 2006) synchronous precursors selection (SPS) MS3 method (method 2) (Schweppe et al., 2020). For method 1, the MS1 scan range was 380–1500 mass-to-charge ratio (*m/z*) and precursor ions were isolated using the quadrupole mass filter with a 0.7 *m/z* isolation window and fragmented with HCD, with a normalised collision energy of 35%. MS2 spectra were acquired in the Orbitrap mass analyser with scan resolution 50,000, maximum injection time 86 ms and AGC target 200%. For method 2, the MS1 scan range was 400–1400 *m/z*. The MS2 spectra were acquired in the ion trap with scan range 140–4000 *m/z* using a rapid scan rate following HCD fragmentation with normalised collision energy of 30%. MS2 spectra were then searched in real time against the UniProt mouse reference proteome (UP000000589) with applied filters as follows – maximum number of peptides per protein, 10; tryptic digest, maximum missed cleavages, 1; maximum search time, 35 ms; static modifications, carbamidomethyl on cysteine and TMTpro16plex on lysine and N-terminus; variable modification, methionine oxidation. Peptide-spectrum matches were selected for SPS MS3 acquisition using multi-notch isolation with ten notches. The selected ions were subject to HCD fragmentation with 55% collision energy, and the SPS MS3 spectra were subsequently obtained using the Orbitrap mass analyser at scan resolution 50,000, scan range 110–500 *m/z*, maximum injection time 120 ms and AGC target 200%. The phosphoproteome was analysed using method 1 and a method 2 incorporating multistage activation (method 3) as described in Jiang et al. (2017). For the MS2 scans, precursor ions were isolated using the quadrupole mass filter with a 0.7 *m/z* window and fragmented using collision-induced dissociation (CID) fragmentation with multistage activation (neutral loss mass, 97.9673) at 35% collision energy, 10 ms activation time and Q parameter for CID activation set to 0.25. MS2 spectra were acquired at a resolution of 30,000 in the Orbitrap mass analyser, with automatic scan range, maximum injection time of 90 ms and AGC target of 200%. The subsequent SPS MS3 spectra followed the same settings as before.

Raw data for the proteome and phosphoproteome were processed separately using MaxQuant v2.1.3.0 (Cox and Mann, 2008; RRID:SCR_014485, www.maxquant.org) and searched against the UniProt mouse reference proteome from March 2021 with peptide-spectrum matches and a protein false discovery rate of 0.01, minimum peptide length of six, two maximum missed cleavages, and reporter ion MS2 or MS3 with correction factors supplied. Cysteine carbamidomethyl was used as a fixed modification, and methionine oxidation, acetyl at protein N-terminus and phosphorylation on serine, threonine or tyrosine (where applicable) were used as variable modifications. Processed data were then analysed using an R-coding script tailored to isobaric labelling mass spectrometry. The script was generated as a hybrid using the backbone and differential gene expression analysis of the ProteoViz package (Storey et al., 2020) as a general script workflow and borrowing the normalization script from the Proteus package (Gierlinski et al., 2018). Briefly, the ‘proteinGroups.txt’ and ‘Phospho (STY)Sites.txt’ tables were read into matrices and filtered for ‘reverse’ hits, ‘potential contaminant’ and proteins ‘only identified by site’. Reporter intensities were corrected for TMT lot number (used for batch-specific isotopic impurity correction factors, supplied by the manufacturer) and normalized using CONSTANd (Van Houtven et al., 2021), log_2_-transformed and differentially analysed using linear models for microarray data (limma; Smyth, 2004). Volcano plots were generated using ggrepel (Wickham, 2016; a ggplot2 extension) as part of the tidyverse.

### Western blot analysis

Cells were washed once with PBS and lysed on ice in RIPA buffer (Millipore, 20-188) containing protease and phosphatase inhibitors (Thermo Fisher Scientific, 78440). Protein levels were normalised using the Pierce BCA protein assay kit as per the manufacturer's instructions. Samples were boiled at 70°C for 15 min in LDS sample buffer (Thermo Fisher Scientific, NP008) and NuPage sample-reducing agent (Thermo Fisher Scientific, NP009). Samples were loaded into 4–12% Bis-Tris gels (Thermo Fisher Scientific, WG1403) and electrophoresis was performed at 100 V for 120 min. The gels were transferred onto a PVDF membrane using an iBlot2 (Thermo Fisher Scientific, IB21001) using program P0. For detection of LRRK2, the gels were transferred onto a PVDF membrane by wet transfer. Membranes were blocked in 5% skimmed milk powder in TBS containing 0.05% Tween 20 (TBS-T) for 1 h at room temperature, then incubated with primary antibody overnight at 4°C. For detection of phosphorylated proteins, membranes were blocked in 5% BSA (Cell Signaling Technology, 9998S) in TBS-T. Membranes were washed in TBS-T and incubated with horseradish peroxidase (HRP)-conjugated secondary antibodies for 1 h at room temperature. Membranes were developed with enhanced chemiluminescence reagent (Bio-Rad) and imaged on an Amersham GE Imager 680 (GE Healthcare). The molecular mass ladder was from Abcam (ab116028). Western blots were quantified by densitometry using FIJI software.

### High-content live imaging of BMDM and iPSDM death

Cells were seeded in 96-well plates (6055302, Revvity) and stained with Blue/Green ReadyProbes Cell Viability Imaging Kit (Invitrogen, R37609) according to the manufacturer's instructions. Cells were treated with 1 mM LLOMe, 300 μg/ml silica or vehicle just prior to the start of imaging. Live-cell snapshot imaging was performed at 37°C and 5% CO_2_ using an automated confocal high-content imaging system (Revvity, Opera Phenix High-Content Screening System) with a 40× 1.1 NA water-immersion objective, excitation lasers (405 nm and 488 nm) and preset emission filters. The 405 nm laser was used at 20% power with 100 ms exposure. The 488 nm laser was used at 5% power with 80 ms exposure. The channels were separated during acquisition to prevent fluorescence crosstalk. Snapshots were taken every 15 or 30 min depending on the length of acquisition and treatment.

Image analysis was performed using Harmony 4.9 image analysis software (Revvity). First, stacks were processed to create a maximum-projection image. The total cell number was calculated using the ‘Find nuclei’ building block in the NucBlue channel (method B). Then, intensity of NucGreen within each nucleus was calculated using the ‘Calculate Intensity Properties’ building block. The total number of dead cells was calculated by setting a threshold for NucGreen intensity and segmenting this population using the ‘Select Population’ building block. The percentage of dead cells was calculated by dividing the total number of NucGreen^+^ nuclei over the total number of NucBlue nuclei.

### Induction of cell death controls and LPS treatment

Where indicated, 250 ng/ml LPS (L8274, Sigma-Aldrich) was added for 3 h prior to downstream applications. For induction of apoptosis, BMDMs were stimulated with 100 ng/ml TNF-α (300-O1A, Peprotech) and 125 nM 5Z-7-oxozeaenol (9890, Sigma-Aldrich) for 7 h. For induction of necroptosis, BMDMs were stimulated with 100 ng/ml TNF-α, 125 nM 5Z-7-oxozeaenol and 10 μM Z-VAD-FMK (tlrl-vad, Invivogen) for 7 h. For induction of pyroptosis, BMDMs were pre-treated with 250 ng/ml LPS for 3 h, followed by 20 μM nigericin (ICT9146, Bio-Rad) for 3 h.

### Flow cytometry

1×10^6^ cells were pelleted by centrifugation (350 ***g*** for 5 min at 4°C), washed with cell-staining buffer (420201, BioLegend) and incubated with TruStain fcX (156604, BioLegend) for 20 min at 4°C. Cells were resuspended in cell-staining buffer with fluorochrome-conjugated antibodies (Table S3) for 30 min at room temperature in the dark. Cells were washed in cell-staining buffer and resuspended in 500 μl cell-staining buffer for acquisition. Data were acquired using a BD LSRFortessa Cell Analyzer (BD Biosciences) and analysed using FlowJo software (v10.8.0). At least 10,000 events per condition were recorded.

### Bafilomycin treatment and DQ-BSA assay

Cells were seeded in 96-well plates (6055302, Revvity) and incubated with 100 nM bafilomycin A1 (Merck, B1793) or vehicle (DMSO) for 2 h. Next, 10 μg/ml DQ-BSA (Thermo Fisher Scientific, D12051) was added for 4 h. The cells were then washed three times in RPMI (BMDMs) or X-VIVO 15 (iPSDMs), counterstained with NucBlue and imaged at 37°C and 5% CO_2_ using an automated confocal high-content imaging system (Revvity, Opera Phenix High-Content Screening System) with a 40×1.1 NA water-immersion objective, excitation lasers (405 nm and 561 nm) and preset emission filters. The 405 nm laser was used at 30% power with 100 ms exposure. The 561 nm laser was used at 20% power with 200 ms exposure.

Image analysis was performed using Harmony 4.9 image analysis software (Revvity). First, stacks were processed to create a maximum-projection image. Cells were segmented using the ‘Find Nuclei’ (method B) and ‘Find Cytoplasm’ (method A) building blocks. Following this, intensity of DQ-BSA in the cytoplasm was calculated using the ‘Calculate Intensity Properties’ building block.

### Lysotracker imaging

Cells were seeded in 96-well plates (6055302, Revvity) and incubated with 25 nM LysoTracker DND-99 (LTR; L7528, Thermo Fisher  Scientific) and NucBlue (Invitrogen, R37609) for 30 min at 37°C and 5% CO_2_. Next, cells were imaged using an automated confocal high-content imaging system (Revvity, Opera Phenix High-Content Screening System) with a 40× 1.1 NA water-immersion objective, excitation lasers (405 nm and 488 nm) and preset emission filters.

Image analysis was performed using Harmony 4.9 image analysis software (Revvity). First, stacks were processed to create a maximum-projection image. Nuclei were segmented using the ‘Find nuclei’ building block (NucBlue channel, method B). Following this, cell boundaries were segmented using the ‘Find cytoplasm’ building block (LTR channel, method A). The cell area was calculated in mm^2^ using the ‘Calculate morphology properties’ building block. Next, the LTR channel image was filtered using ‘Texture SER’ and ‘SER Bright’ methods (2 mm, unnormalized) to highlight the brightest portion of the LTR^+^ spots. Following this, LTR^+^ spots were segmented using the ‘Find image region’ building block (common threshold 0.35) and the mean LTR^+^ cytoplasm intensity and LTR^+^ spot area were calculated.

### Statistical analyses

Statistical analyses were performed using GraphPad Prism software version 9.3.1. Data are shown as mean±s.d. Unpaired two-tailed *t*-tests were performed for statistical analysis of experiments with two groups. For more than two groups, analysis was performed using one-way ANOVA with Šidák's multiple comparisons test or two-way ANOVA with Šidák's multiple comparisons test, as indicated in the figure legends.

## Supplementary Material

10.1242/joces.265083_sup1 Supplementary information
