# Supplementary material for: The LRRK2 G2019S variant rewires Rab GTPase phosphorylation after lysosomal damage and promotes cell death in human macrophages
Source: J Cell Sci. 2026 Jul 27;139(14):jcs265083. doi: 10.1242/jcs.265083 (PMC13490737; doi:10.1242/jcs.265083)
Supplement: Supplementary file 1 [file joces-139-265083-s1.pdf]

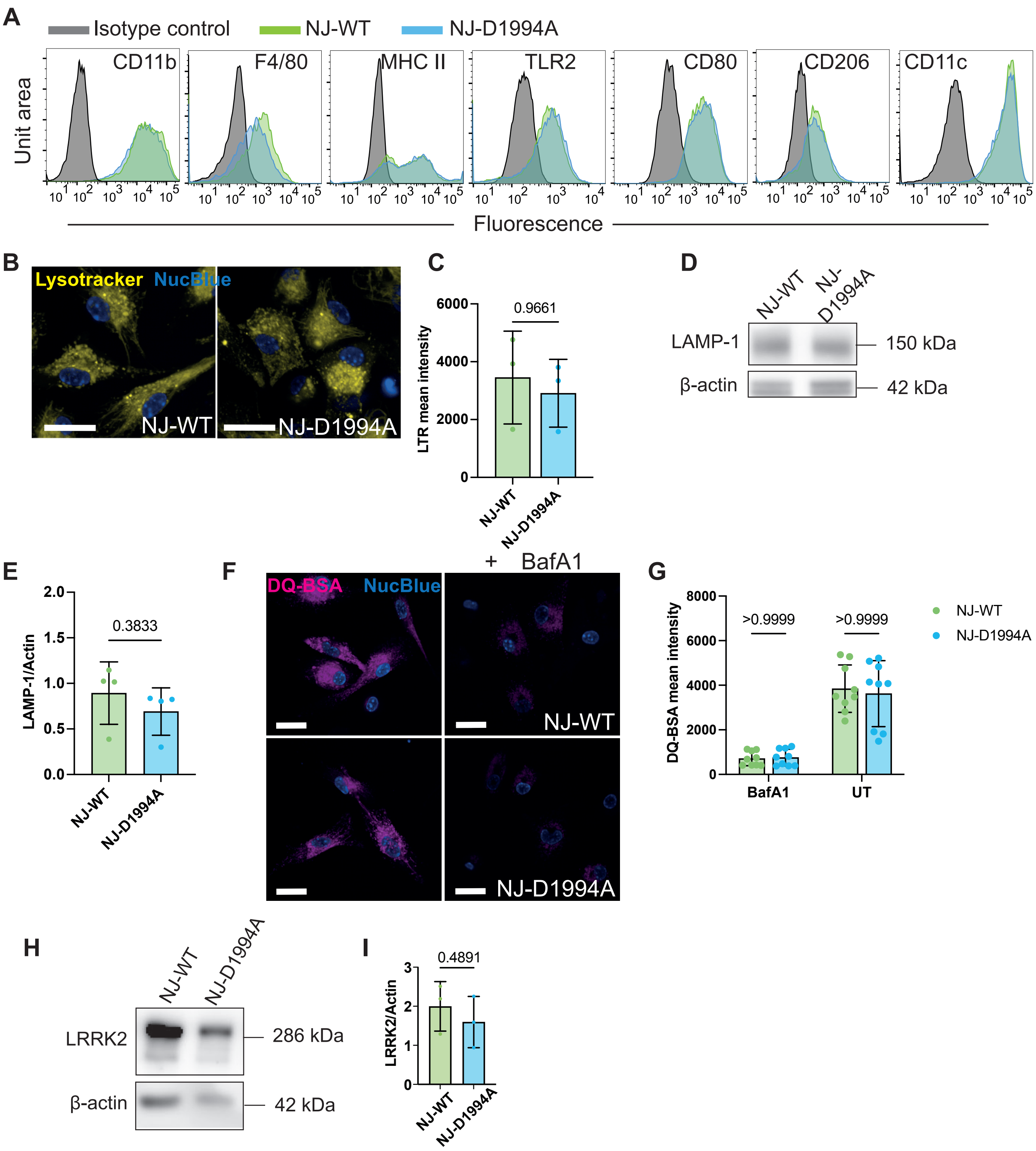

Fig. S1. Immunophenotyping and characterisation of lysosomal activity from NJ-WT and NJ-D1994A macrophages. (A) Flow cytometry characterisation of surface expression of macrophage markers. Data are from one representative experiment. Data are presented as histograms with compensated fluorescence of the indicated surface marker on the x-axis. (B) Representative images of live NJ-WT and NJ-D1994A macrophages stained with Lysotracker (LTR) and NucBlue dye. Scale bars = 20 µm. (C) Quantitative analysis of the mean LTR cytoplasmic intensity. Data shown is mean ± SD from 3 independent experiments (n=5 independent wells). One-way ANOVA followed by Šidák's multiple comparisons test. (D) Western blot analysis of LAMP-1 and β-actin. (E) LAMP-1 band intensity was quantified by densitometry and normalised to β-actin. Data are mean ± SD from 4 independent experiments. Two-tailed t-test. (F) Representative images of live cells pre-treated with Bafilomycin A1 (BafA1) 100 nM followed by incubation with DQ-BSA and NucBlue dye. Scale bars = 20 µm. (G) Quantitative analysis of the mean DQ-BSA cytoplasmic intensity. Data shown is mean ± SD from 3 independent experiments (n=9 independent wells). Two-way ANOVA followed by Šidák's multiple comparisons test. (H) Western blot analysis of LRRK2 and β-actin. (I) LRRK2 band intensity was quantified by densitometry and normalised to β-actin. Data are mean ± SD from 3 independent experiments. Two-tailed t-test.

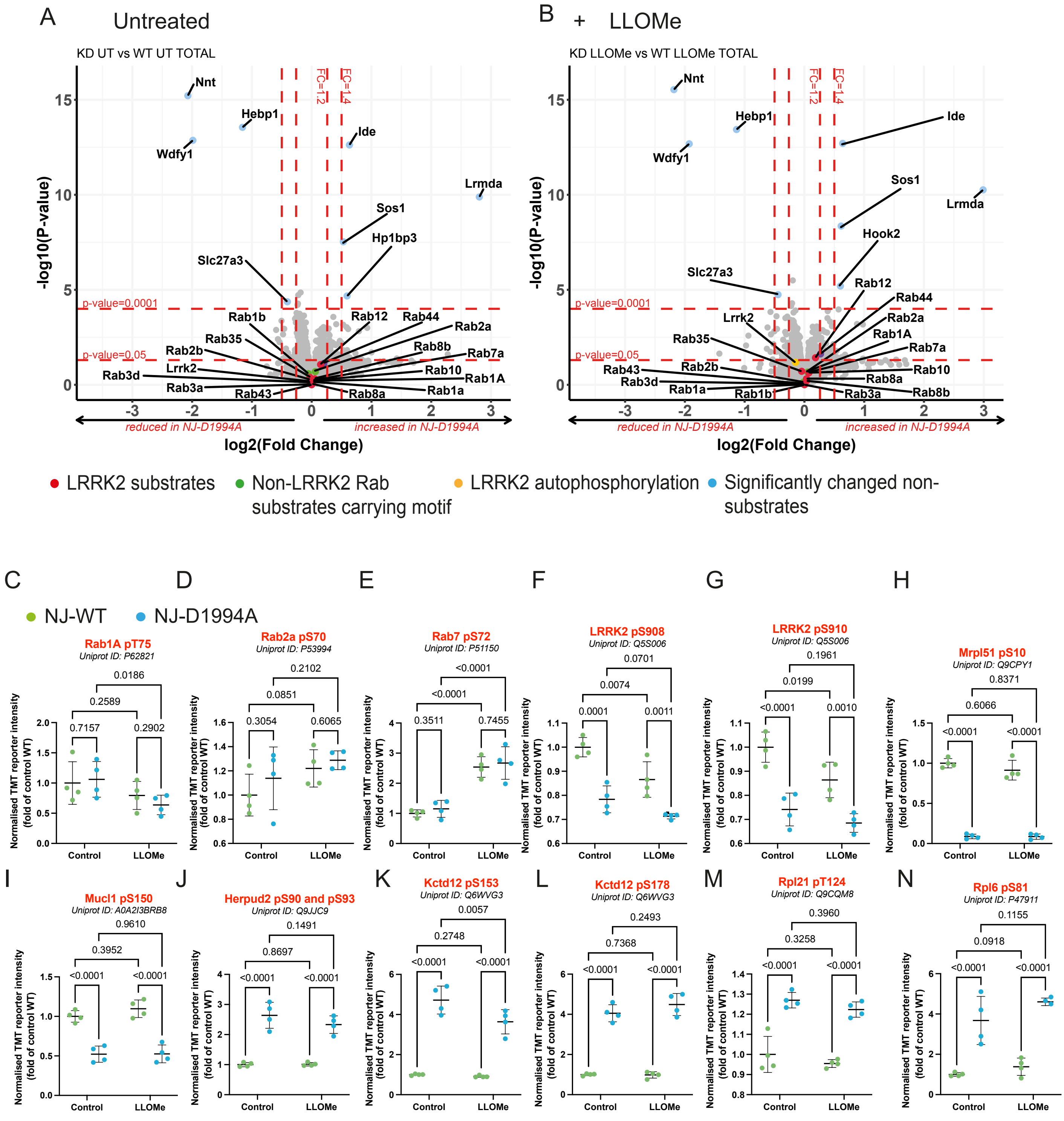

Fig. S2. Proteomic and phosphoproteomic analysis in NJ-WT and NJ-D1994A macrophages. (A and B) Macrophages were treated with LLOMe 1mM for 30 minutes and cells were analysed by TMTpro 16-plex mass spectrometry. Volcano plots of the total proteome in the untreated (A) and LLOMe-treated (B) conditions of the difference in total protein levels between NJ-WT and NJ-D1994A macrophages. Each point represents one protein. The x-axis shows the log2-transformed fold change, and the y-axis shows the significance by -log10-transformed P-value, obtained by linear models for microarray data. A fold-change greater than 1.2 and p value < 0.05 was deemed significant. (C to N) Scatterplots showing the normalised TMT reporter intensity data obtained by mass spectrometry and p-values for: non-LRRK2 Rab substrates carrying the switch II motif (C to E); LRRK2 phosphorylation sites pS908 and pS910 (F and G); significantly altered non-substrates (H to N); (J) Serines 90 and 93 on Herpud2 are phosphorylated simultaneously and their quantifications are derived from the same doubly phosphorylated peptide. CONSTANd-normalised and log2-transformed raw TMT reporter intensities were back transformed (2^x) and expressed relative to the mean of the control WT group (set at 1). N=4 biological replicates per condition. P values by linear models for microarray data (limma) statistics on the normalised log2-transformed data. Error bars represent SD.

Journal of Cell Science • Supplementary information

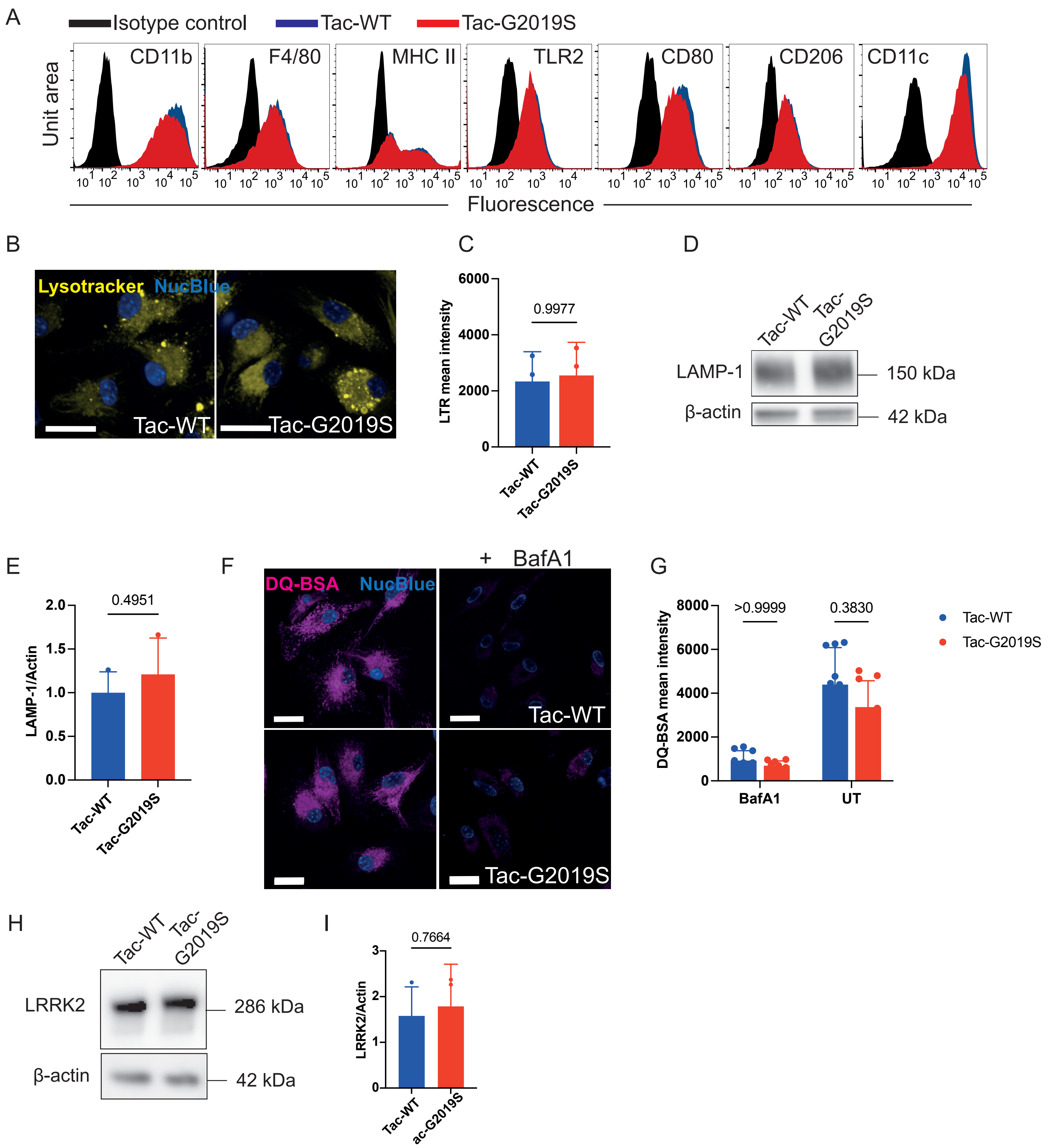

Fig. S3. Immunophenotyping and characte risation of lysosomal activity from Tac-WT and Tac-G2019S macrophages. (A) Flow cytometry characterisation of surface expression of macrophage markers. Data are from one representative experiment. Data are presented as histograms with compensated fluorescence of the indicated surface marker on the x-axis. (B) Representative images of live Tac-WT and Tac-G2019S macrophages stained with Lysotracker (LTR) and NucBlue dye. Scale bars = 20 μm. (C) Quantitative analysis of the mean LTR cytoplasmic intensity. Data shown is mean ± SD from 3 independent experiments. Two-tailed t-test. (D) Western blot analysis of LAMP-1 and β-actin. (E) LAMP-1 band intensity was quantified by densitometry and normalised to β-actin. Data are mean ± SD from 3 independent experiments. Two-tailed t-test. (F) Representative images of live cells pre-treated with Bafilomycin A1 (BafA1) 100 nM followed by incubation with DQ-BSA and NucBlue dye. Scale bars = 20 μm. (G) Quantitative analysis of the mean DQ-BSA cytoplasmic intensity. Data shown is mean ± SD from 3 independent experiments (n=9 independent wells). Two-way ANOVA followed by Šidák's multiple comparisons test. (H) Western blot analysis of LRRK2 and β-actin. (I) LRRK2 band intensity was quantified by densitometry and normalised to β-actin. Data are mean ± SD from 3 independent experiments. Two-tailed t-test.

Journal of Cell Science • Supplementary information

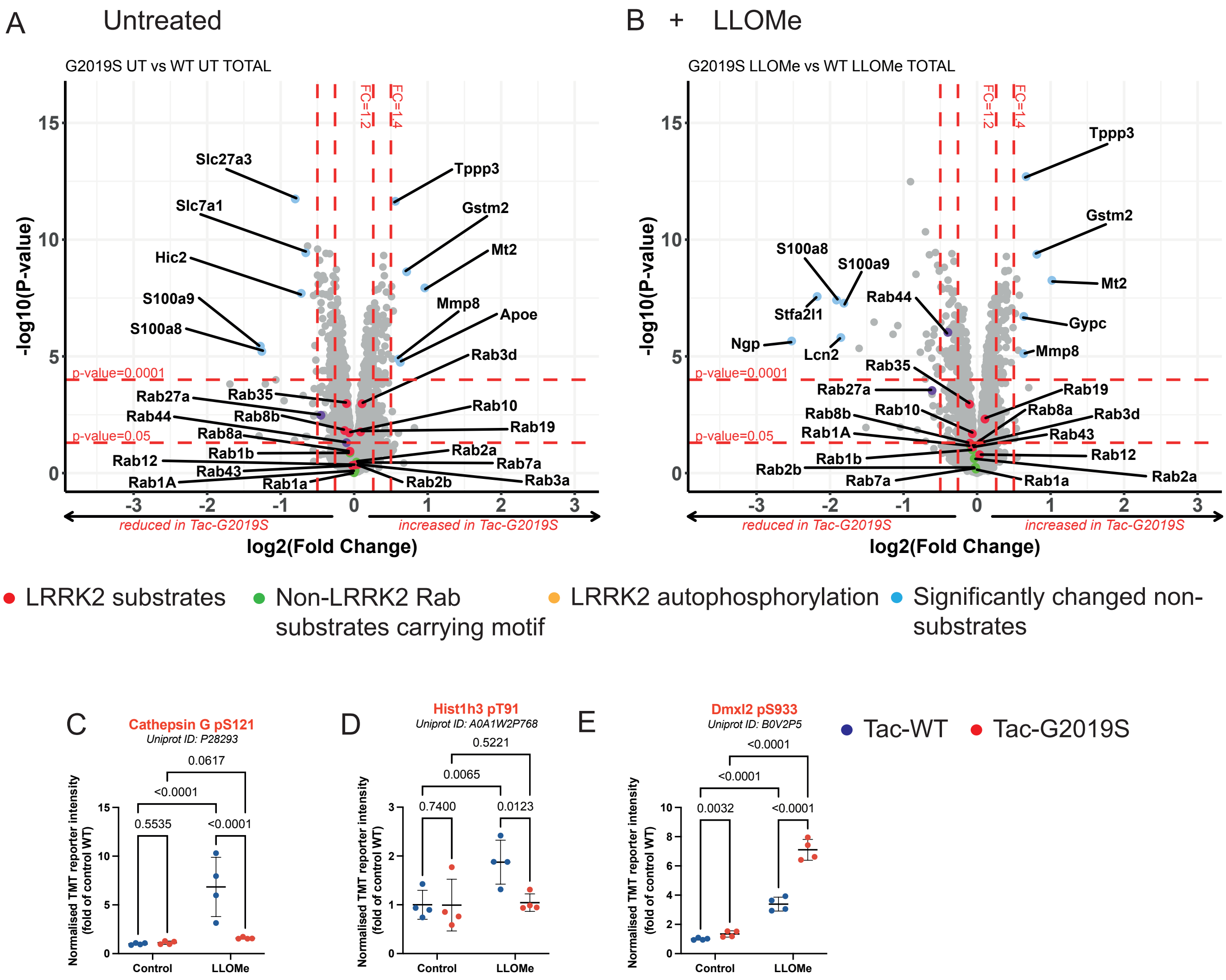

Fig. S4. Proteomic and phosphoproteomic analysis in Tac-WT and Tac-G2019S macrophages. (A and B) Macrophages were treated with LLOMe 1 mM for 30 minutes and cells were analysed by TMTpro 16-plex mass spectrometry. Volcano plots of the total proteome in the untreated (A) and LLOMe-treated (B) conditions of the difference in total protein levels between Tac-WT and Tac-G2019S macrophages. Each point represents one protein. The x-axis shows the log2-transformed fold change, and the y-axis shows the significance by -log10-transformed P-value, obtained by linear models for microarray data. A fold-change greater than 1.2 and p value<0.05 was deemed significant. (C to E) Scatterplots showing the normalised TMT reporter intensity data obtained by mass spectrometry and p-values for significantly altered non-substrates. CONSTANd-normalised and log2-transformed raw TMT reporter intensities were back-transformed (2^x) and expressed relative to the mean of the control WT group (set at 1). N = 4 biological replicates per condition. P values by linear models for microarray data (limma) statistics on the normalised log2-transformed data. Error bars represent SD.

Journal of Cell Science • Supplementary information

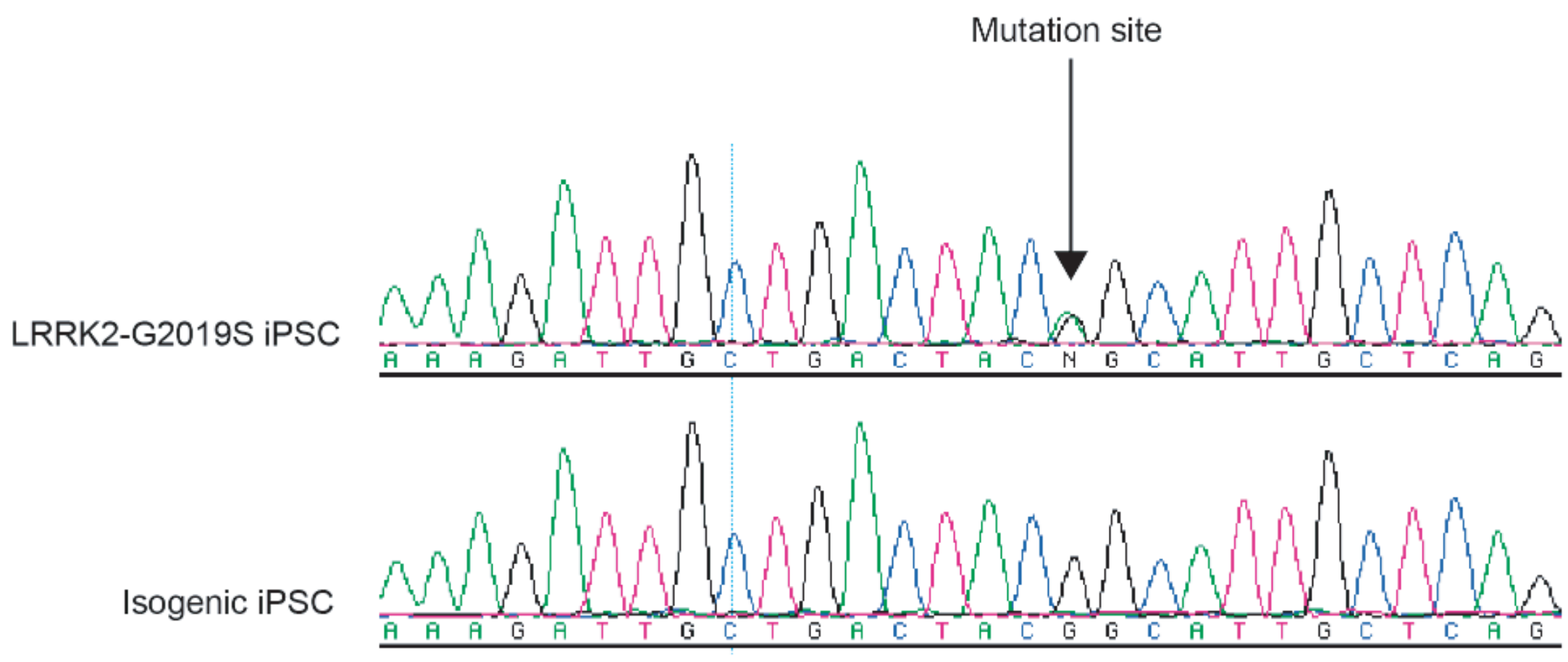

Fig. S5. Sequencing results of isogenic clone after genome editing of LRRK 2019S iPSC. Sanger sequencing chromatograms of LRRK2 G2019S iPSC (clone ID: CDI00002173) and the corresponding isogenic control clone (0043052321) generated by CRISPR/Cas9-mediated genome editing. The region encompassing the G2019S mutation (c.6055G>A, p.Gly2019Ser) is shown. The isogenic clone shows restoration of the wild-type guanine at position c.6055, confirming successful correction of the pathogenic variant. Arrows indicate the position of the edited nucleotide.

Journal of Cell Science • Supplementary information

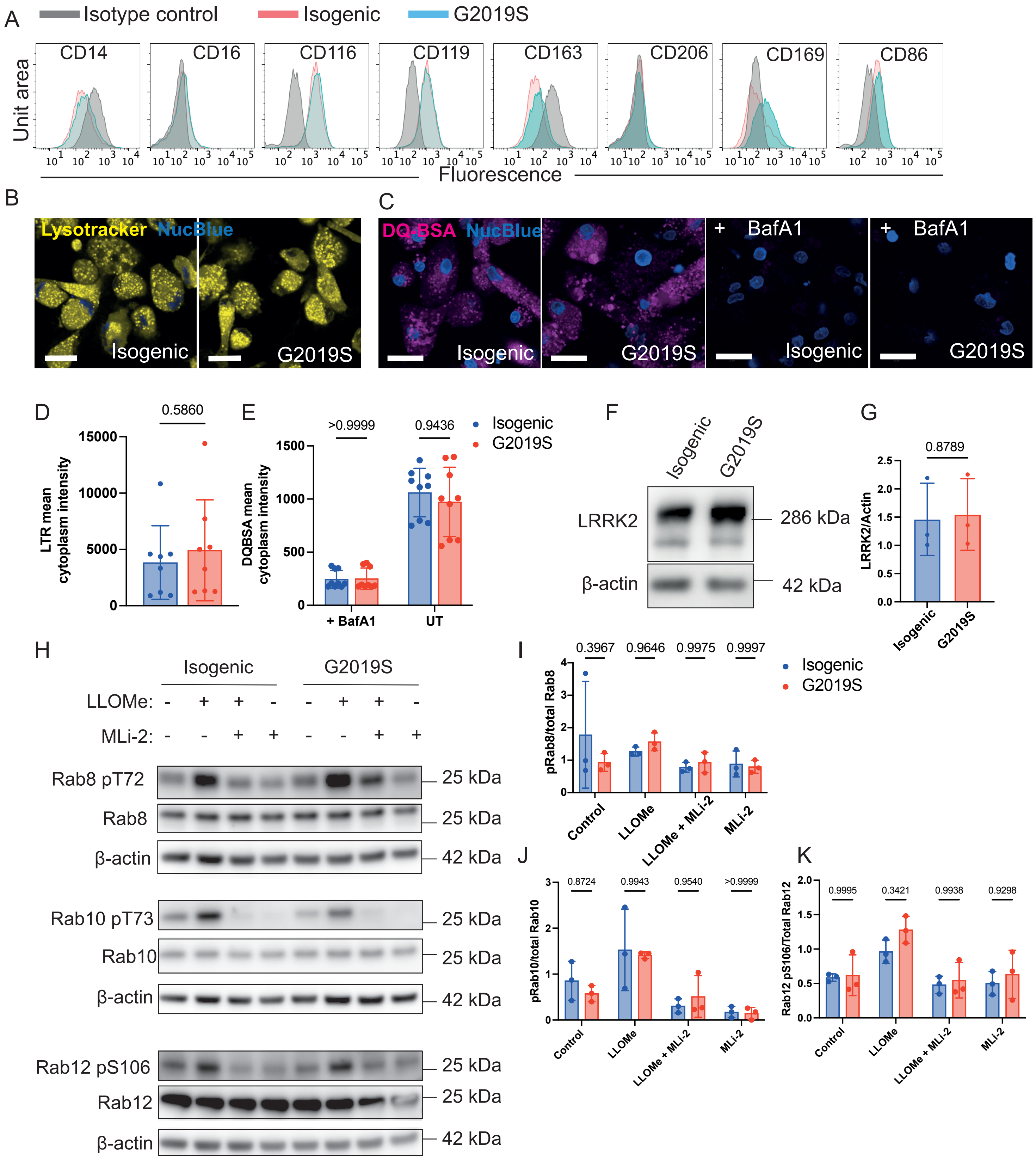

Fig. S6. Immunophenotyping and characterisation of lysosomal activity from LRRK2-G2019S and Isogenic control human iPSC-derived macrophages. (A) Flow cytometry characterisation of surface expression of macrophage and monocyte markers in induced pluripotent stem cell induced macrophages (iPSDM). Data are from one representative experiment. Data are presented as histograms with compensated fluorescence of the indicated surface marker on the x-axis. (B) Representative images of live iPSDM stained with Lysotracker (LTR) and NucBlue dye. Scale bars = 20 µm. (C) Representative images of live iPSDM pre-treated with Bafilomycin A1 (BafA1) 100 nM followed by incubation with DQ-BSA and NucBlue dye. Scale bars = 20 µm. (D) Quantitative analysis of the mean LTR cytoplasmic intensity. Data shown is mean ± SD from 3 independent experiments. Two-tailed t-test. (E) Quantitative analysis of the mean DQ-BSA cytoplasmic intensity. Data shown is mean ± SD from 3 independent experiments. Two-way ANOVA followed by Šidák's multiple comparisons test. (F) Western blot analysis of LRRK2 and β-actin. (G) LRRK2 band intensity was quantified by densitometry and normalised to β-actin. Data are mean ± SD from 3 independent experiments. Two-tailed t-test. (H) Western blot analysis of Rab8 pT72, Rab8, Rab10 pT73, Rab10, Rab12 pS105, Rab12 and β-actin in isogenic and G2019S iPSDM in untreated, MLi-2, LLOMe and LLOMe + MLi-2 conditions. (B to D) Rab8 pT72, Rab10 pT73 and Rab12 pS105 band intensities were quantified by densitometry and normalised to Rab8, Rab10 and Rab12, respectively. Data are mean ± SD from 3 independent experiments. Two-way ANOVA followed by Šidák's multiple comparisons test.

Journal of Cell Science • Supplementary information

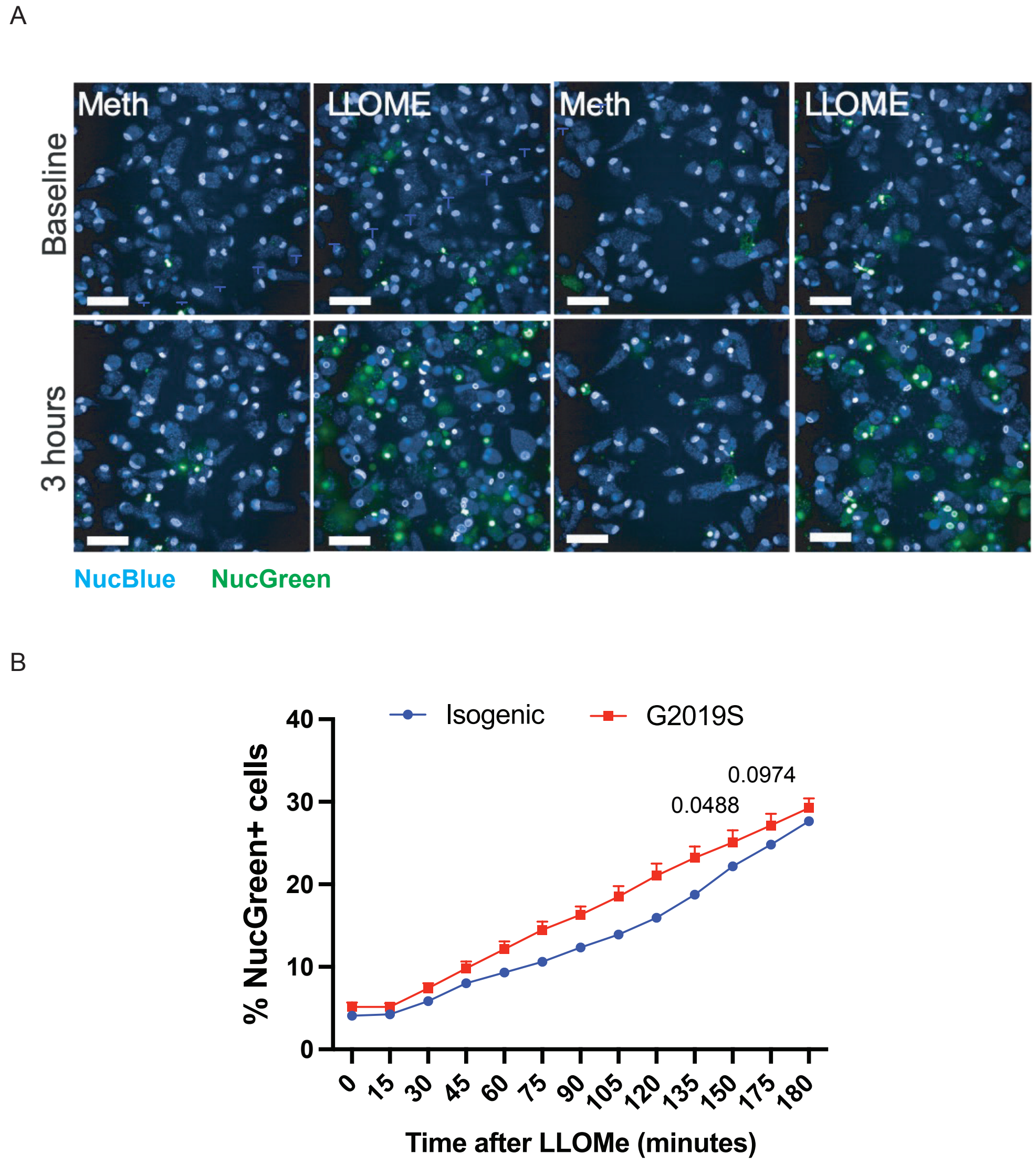

Fig. S7. LRRK2 G2019S increases susceptibility to lysosomal damage–induced cell death in a second clone of patient-derived induced pluripotent stem cell-derived macrophages. (A) Snapshot of live iPSDM (clone ID: CDI00008375 and isogenic) treated with LLOMe 1 mM and imaged every 15 minutes. Nuclear staining for live/dead (blue/green). Scale bars = 50 µm. (B) Quantitative analysis of the percentage of dead cells at each timepoint. Data shown is the mean ± SD from 2 independent experiments with 6 technical replicates. Two-way ANOVA followed by Šidák's multiple comparisons test.

Table S1. Sequences of sgRNAs and ssODNs used in the study.

**Name**	**Sequencing 5'->3'**
LRRK2-1step	TGCTCAGTACTGCTGTAGAATGG
LRRK2-2step<br>(CDI00002173)	TGTAGTCAGCAATCTTTGCA
ssODN-LRRK2	GAAACCCCACAATGTGCTGCTTTTCACACT<br>GTATCCCAATGCTGCCATCATTGCAAAGA<br>TTGCTGACTACGGCATTGCTCAGTACTGCTGTAGAATGGGG

Table S2. Primers for iPSC genotyping.

**Primer name**	**Sequence**
**Primers for PCR**	
G2019S_GR1	5' – TAT ATC TCC TAG ACC CAC ACT TGA G – 3'
G2019S_GF1	5' – TAC CAG GCT TGA TGC TTT AGT TAT G -3'
**Primers for sequencing**	
G2019S_SR1	5' – GGG CAC TGA TGG TCC ACT GC -3'
G2019S_SF1	5' – TGG CAG GTA TCT CCA CTC AG -3'

Journal of Cell Science • Supplementary information

Table S3. Primary antibodies used for western blot, immunofluorescence, and flow cytometry.

Target	Host species	Company	Catalogue number	Dilution	Application
Rab8	Rabbit	Cell Signaling	6975	1:1000	Western blot
Rab8 pT72	Rabbit	Abcam	ab230260	1:1000	Western blot
Rab10	Rabbit	Cell Signaling	8127	1:1000	Western blot
Rab10 pT73	Rabbit	Abcam	ab230261	1:1000	Western blot
Rab12	Rabbit	ProteinTech	18843	1:1000	Western blot
Rab12 pS105	Rabbit	Abcam	ab256487	1:1000	Western blot
Cleaved caspase-3 (Asp175)	Rabbit	Cell Signaling	9664T	1:1000	Western blot
Phospho-RIP3 (Thr231/Ser232)	Rabbit	Cell Signaling	91702	1:1000	Western blot
Cleaved Gasdermin D (Asp275)	Rabbit	Cell Signaling	10137	1:1000	Western blot
Cleaved PARP (Asp214)	Rabbit	Cell Signaling	94885	1:1000	Western blot
Phospho-MLKL (Ser 345)	Rabbit	Cell Signaling	37333S	1:1000	Western blot
Cleaved-IL-1β (Asp117)	Rabbit	Cell Signaling	63124S	1:1000	Western blot
LAMP1	Rat	DSHB	1D4B	1:1000	Western blot
β-Actin-HRP	Mouse	Cell Signaling	12262S	1:5000	Western blot
LRRK2	Rabbit	Abcam	Ab133474	1:1000	Western blot
F4/80	Rat	BioLegend	123113	1:80	Flow cytometry
CD11b	Rat	Biolegend	101237	1:20	Flow cytometry
MHCII	Rat	Biolegend	107607	1:80	Flow cytometry
CD11c	Armenian Hamster	BioLegend	117319	1:200	Flow cytometry
TLR2	Mouse	BioLegend	153009	1:20	Flow cytometry
CD80	Armenian Hamster	BioLegend	104717	1:200	Flow cytometry
CD206	Rat	BioLegend	141717	1:20	Flow cytometry
CD14	Mouse	BD Biosciences	562689	1:20	Flow cytometry
CD16	Mouse	BD Biosciences	557710	1:20	Flow cytometry
CD119	Mouse	BD Biosciences	558934	1:50	Flow cytometry
CD163	Mouse	BD Biosciences	563697	1:20	Flow cytometry
CD206	Mouse	BD Biosciences	561763	1:5	Flow cytometry
Siglec-1 (CD169)	Mouse	BD Biosciences	565248	1:20	Flow cytometry
CD86	Mouse	BD Biosciences	562433	1:20	Flow cytometry

Journal of Cell Science • Supplementary information
